# Mechanochemistry Meets Catalysis: Metal Complexes for Greener Organic Transformations

**DOI:** 10.1002/anie.202600005

**Published:** 2026-05-23

**Authors:** Sourav Behera, Francesco Basoccu, Andrea Porcheddu

**Affiliations:** ^1^ Dipartimento di Scienze Chimiche e Geologiche, Università degli Studi di Cagliari Cittadella Universitaria Monserrato Italy

**Keywords:** mechanocatalysis, mechanochemistry, mechano‐redox, transition metal complexes

## Abstract

Mechanochemistry and transition‐metal catalysis are converging into a platform in which mechanical energy reshapes catalyst formulation, speciation, and operative state, rather than simply replacing solvent. Through intense mixing, continuously renewed interfaces, liquid‐assisted grinding (LAG), and rheological control, milling can direct metal‐complex assembly, activation, reactivity, and selectivity in ways difficult to reproduce in solution. These attributes streamline catalyst preparation, lessen dependence on stringent inert‐atmosphere protocols, and open access to transformations that are inefficient, selective only under milling, or inaccessible by conventional methods. This Review examines the mechanochemical synthesis of transition‐metal complexes and their direct deployment in catalytic organic transformations, from earth‐abundant first‐row metals to selected noble‐metal systems. Quantitative benchmarks, including enantioselectivities up to 99% ee, turnover frequencies above 100 h^−1^, and cross‐electrophile couplings completed within minutes, demonstrate that mechanocatalysis can deliver not only greener variants of known reactions but also distinct reactivity regimes. Mechanistic uncertainty, reproducibility, and scalable technologies such as twin‐screw extrusion (TSE) and resonant acoustic mixing (RAM) are assessed, framing mechanocatalysis as both an enabling methodology and a conceptual basis for next‐generation green catalysis.

## Graphical abstract

Mechanochemistry is redefining metal catalysis by controlling catalyst formulation, speciation, and deployment. This Review shows how milling, LAG, RAM, and TSE enable rapid metal‐complex assembly, distinctive catalytic manifolds, and scalable synthesis beyond solution chemistry.

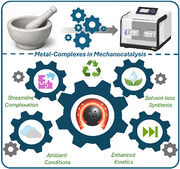

## 1 Introduction

Transition‐metal catalysis underpins modern synthesis, but many protocols remain solvent‐intensive, dilute, atmosphere‐sensitive, and operationally demanding despite major advances in catalyst and ligand design [1, 2, 3, 4, 5, 6, 7, 8, 9, 10, 11, 12, 13, 14]. Sustainable catalysis therefore requires not only better catalysts and greener feedstocks, but process platforms that reduce solvent dependence, intensify mass transfer, and compress workflows without compromising performance.

Mechanochemistry addresses these goals by delivering energy through impact and shear, maintaining intimate contact between solid reagents, metal precursors, ligands, bases, and additives in the absence (or near absence) of bulk solvent [15, 16]. Mechanochemical activation can blur or even remove the operational boundaries that typically separate ligand formation, salt metathesis, catalyst assembly, and catalytic turnover in a solution‐phase workflow. This can shorten sequences, simplify handling, reduce reliance on inert conditions, and unlock sluggish, inhibited, or impractical reactivity in conventional media [15, 16, 17].

**FIGURE 1:**
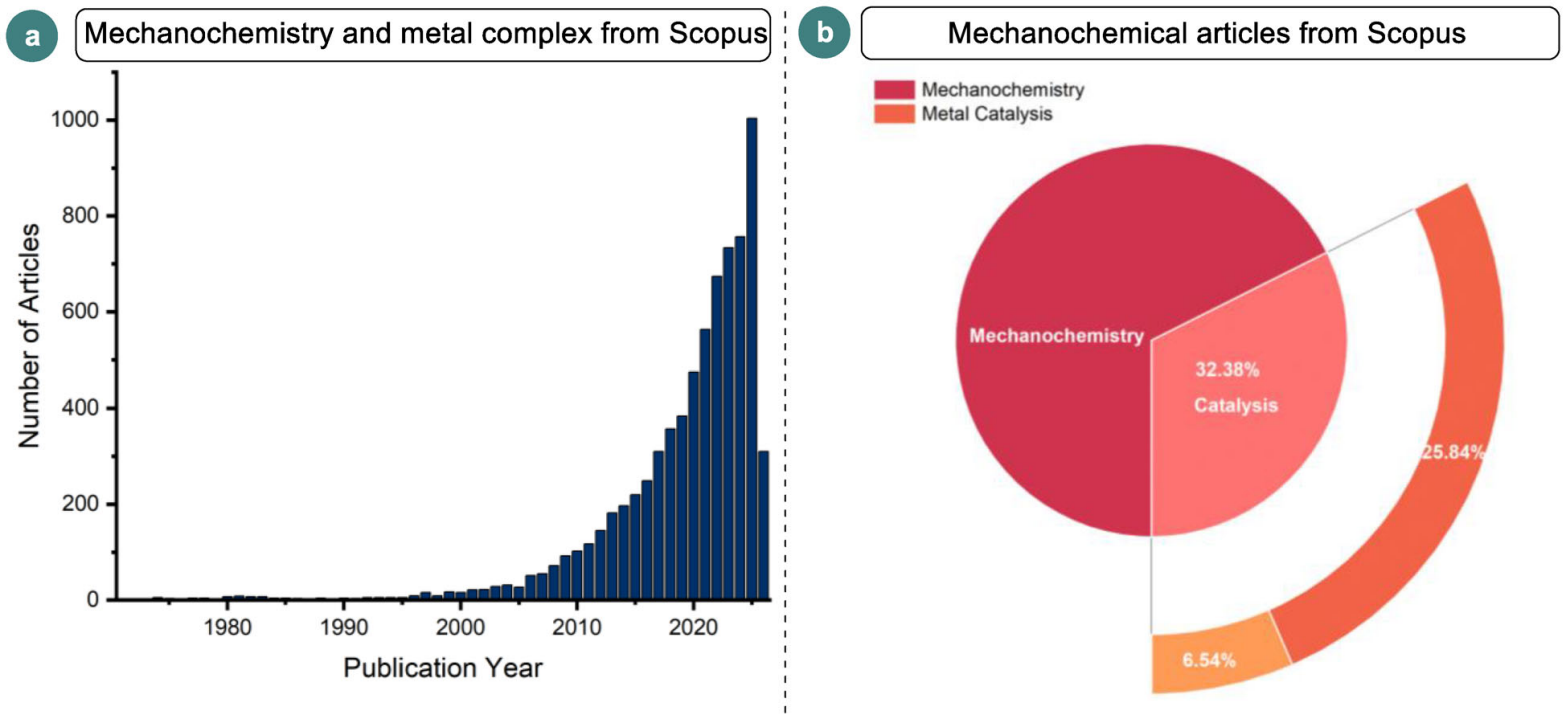
(a) Annual number of publications retrieved from Scopus using the keywords ‘mechanochemistry’ and ‘metal complex’ (7341 records, accessed March 27, 2026). (b) Share of records containing the keywords ‘mechanochemistry’ and ‘metal catalysis’ relative to the overall mechanochemistry literature indexed in Scopus from 1960 to 2026 (28,933 records on mechanochemistry; 25.84% of the literature features ‘mechanochemistry’ and ‘metal catalysis’; accessed March 27, 2026).

Mechanochemistry has evolved from a solvent‐free alternative into an enabling paradigm that aligns with the 12 Principles of Green Chemistry while offering operational advantages, including reduced solvent demand, intensified kinetics, simplified workflows, and stabilization of reactive species in solid formulations [15, 16, 17, 18, 19, 20, 21]. Simultaneously, mechanochemistry introduces a distinctive design space in which the “*energy delivery mode*” and formulation (rather than only the molecular structure in solution) become tunable variables. This shift is particularly consequential for metal complexes, whose speciation, coordination environment, and catalytic competence may depend strongly on the local composition, microstructure, and phase evolution. As the literature has expanded, the combined themes of (i) the mechanochemical preparation of metal complexes and (ii) their deployment as catalysts have emerged as a coherent and rapidly developing intersection that is now sufficiently mature to support general principles rather than isolated demonstrations (Figure 1).

This Review centers on that convergence and asks a pragmatic question: when does mechanochemistry offer a distinctive advantage for metal‐complex synthesis and catalysis, and why? Answering this requires treating mechanochemistry as both a reactivity platform and a process technology [22].

### 1.1 From Metal Complexes to Catalyst Formulations

Within the mechanochemical landscape, metal‐mediated mechanocatalysis has become one of the most developed domains, where the catalyst may be a solid complex, a supported species, a transient intermediate, or an active surface generated and renewed by mechanical action [23]. From Theophrastus’ trituration of cinnabar with copper and vinegar [24] to modern solvent‐free synthesis [25], mechanochemistry has evolved from empirical practice into a platform for green synthesis. Its extension to coordination compounds is natural: complex formation depends on productive contact between metal precursors, ligands, counterions, bases, and additives. Milling enforces this contact under concentrated, solvent‐minimized conditions, accelerating ligand exchange, salt metathesis, deprotonation–coordination events, and assembly pathways that are slow or equilibrium‐limited in solution. It can also reshape speciation by biasing local stoichiometry, microenvironment, and phase behavior, occasionally stabilizing coordination motifs difficult to access reproducibly in solution [26, 27, 28, 29]. Operationally, these features enable either the isolation of robust solid complexes or the in situ generation of short‐lived catalytically competent species, avoiding purification, transfer, and handling steps that increase waste and reduce robustness.

Mechanochemistry, therefore, shifts attention from the “molecule in solvent” to the “catalyst formulation”. Particle size, dispersion, additives, and templating solids influence phase formation, active‐site accessibility, and microscale heat and mass transfer. Catalyst structure and catalyst state, including phase and microstructure, can thus be engineered together with an operational simplicity difficult to reproduce in solution [26, 27, 28, 29].

### 1.2 Beyond Solvent Replacement: Variables, Platforms, and Control

The same feature that makes mechanochemistry powerful also complicates translation: outcomes are highly sensitive to how mechanical energy is delivered. Frequency, time, reactor geometry, media composition, atmosphere, temperature, and grinding auxiliaries can all imprint kinetics, selectivity, and solid‐state evolution. This complexity is a design space, but making it transferable requires clearer protocols, standardized reporting, and quantitative descriptors of mechanical intensity.

Liquid‐assisted grinding (LAG) [30] exemplifies this design space [30]. Trace liquids modulate surface mobility, wetting, local solvation, and rheology, thereby influencing rate, selectivity, polymorphism, crystallinity, phase evolution [30], and, in coordination chemistry, metal speciation, ligand arrangement, nuclearity, and topology. LAG should therefore be treated as a chemically meaningful variable rather than an empirical additive [31].

At the process level, twin‐screw extrusion (TSE) and resonant acoustic mixing (RAM) have been instrumental in moving mechanochemistry beyond laboratory‐scale milling toward scalable manufacturing. TSE [32], in particular, provides continuous control over energy input, residence time, temperature, and material throughput under solvent‐free or solvent‐lean conditions, and has become a defining platform for the transition from mechanochemistry as a laboratory technique to mechanochemistry as a manufacturing strategy [32].

In parallel, RAM has emerged as a complementary, media‐free technology capable of delivering intense mixing without milling balls. By minimizing contamination risks, simplifying reactor design, and preserving scalability, RAM expands the operational landscape of mechanochemical processing beyond conventional impact‐ and attrition‐based millin [30, 33, 34, 35].

Real‐time interrogation is becoming central to predictability. In situ diffraction, spectroscopy, temperature and pressure tracking, and other process‐aware measurements are moving the field beyond endpoint analysis and empirical optimization [36]. This is especially important for metal‐complex synthesis and catalysis, where the active species may depend on time‐dependent speciation and phase evolution. Mechanochemistry should therefore be viewed not merely as a greener alternative, but as a complementary synthetic paradigm governed by energy delivery, formulation, and phase evolution [37, 38, 39, 40, 41].

### 1.3 Quantifying Mechanical Input: From Parameters to Predictive Descriptors

Despite its synthetic power, mechanochemistry lacks the quantitative vocabulary that has transformed solution‐phase catalysis from empirical practice into predictive science. Parameters routinely reported in ball‐milling studies, namely milling frequency, time, jar material, ball size, and reaction scale, define the experiment operationally, but say comparatively little about how mechanical input is partitioned, dissipated, and converted into chemically productive events. They describe conditions rather than causality.

Early efforts to rationalize this conversion, most notably the “hot spot” model [42], provided an important starting point by attributing reactivity to highly localized, short‐lived temperature spikes generated during collision events. Evidence from in situ monitoring [43] and computational studies [44, 45, 46] indicates that ball‐milling reactivity cannot be reduced to a purely thermal phenomenon. Instead, it arises from a coupled interplay of impact dynamics, stress accumulation, defect formation, particle size evolution, changing interfaces, and phase reorganization. The central challenge is to translate these variables into quantitative, transferable descriptors. Neither atomistic simulation nor in situ monitoring alone provides a general solution: simulations remain demanding, whereas in situ methods are often instrument‐specific. Kinematic models, therefore, offer an intermediate framework by linking measurable parameters, including ball mass, milling frequency, and jar geometry, to descriptors of impact and energy transfer [47, 48].

**FIGURE 2:**
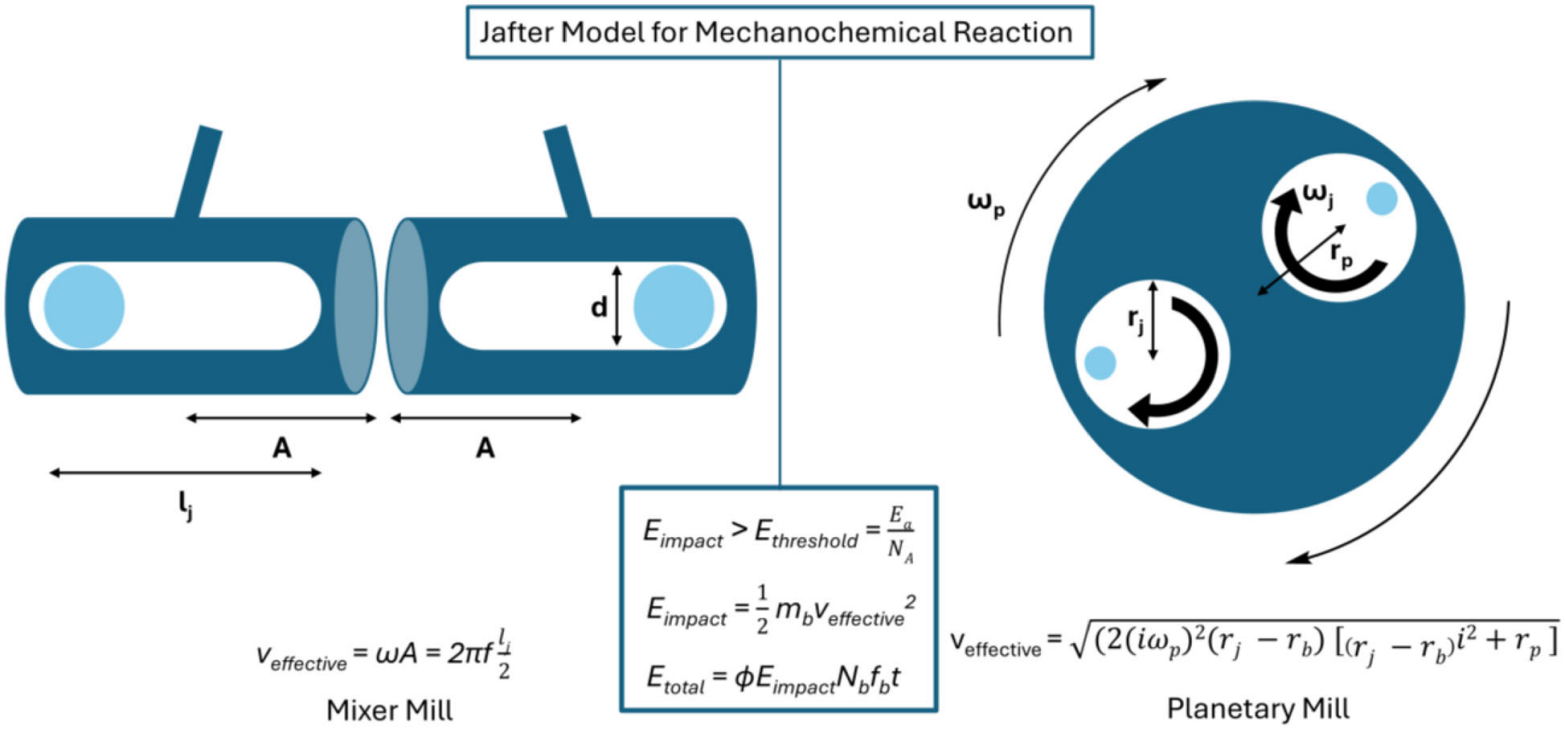
Schematic representation of the energy‐budget framework governing mechanochemical reaction, as proposed by Jafter and coworkers [49].

Within this context, the “energy budgeting” model introduced by Jafter and coworkers represented a major conceptual advance (Figure 2) [49]. Its central premise is deceptively simple: a mechanochemical event becomes productive only when the energy delivered in a single collision, *E*_impact_, exceeds the activation threshold, *E*_threshold_. Once this condition is satisfied, the reaction progress can be correlated with the cumulative energy input E_total_. By formulating distinct treatments for mixer mills and planetary mills, the model showed that comparable conversions can be achieved when similar *E*_total_ values are delivered, even when the milling parameters and, in some cases, the mill type differ substantially. This behavior has been demonstrated across several transformations, including amidation [50], Suzuki coupling [51], Grignard addition [52], and porphyrin metalation [53], establishing cumulative energy as a valuable first‐order descriptor of mechanochemical performance.

The strengths of this framework also expose its limits: once conversion is complete, further milling only inflates *E*_total_, while much of the applied input is dissipated as heat, sound, or vessel deformation. Aging, transient temperature excursions, and liquid additives are therefore not secondary complications, but variables that reshape how energy becomes reactivity [54]. The model remains a useful baseline and has been reproduced upon scale‐up by up to one order of magnitude.

In mechanochemical Finkelstein halogen exchange, Templ and coworkers found that protocols delivering the same *E*_total_ of 12,000 J led to markedly different outcomes depending on the energy‐delivery method [55]. Lower‐frequency, longer‐duration conditions consistently outperformed higher‐frequency, shorter‐duration conditions, with yield gains of up to 60%. These results argue against a purely cumulative picture and instead point to an impact‐governed regime in which the decisive parameter is the energy of the individual collision, which is strongly dependent on ball mass and density. In other words, not all joules are mechanochemically equivalent in this context.

LAG follows the same logic: its response is often nonlinear. A defined optimum, for example, *η* = 0.5 µL mg^−1^ in Wittig olefinations, indicates that small amounts of liquid improve contact without compromising impact efficiency, whereas excess liquid dampens collision dynamics [54]. Aging adds a temporal dimension, confirming that performance depends not only on energy input, but on how that energy is delivered and dissipated.

Mechanochemical reactions are unlikely to obey a single universal energetic law. Some appear to be controlled primarily by cumulative input, others by single‐impact thresholds, and others by a more intricate interplay of impact, time, medium, and evolving material properties. The field therefore requires a discriminating quantitative framework capable of identifying the operative energetic regime and moving mechanochemistry beyond protocol‐driven optimization toward predictive design.

Perhaps the most provocative concept to emerge from recent mechanochemical theory is the activation‐barrier paradox advanced by Borchardt and coworkers [56]. Contrary to the widespread assumption that milling necessarily lowers the activation barrier [57], several solid‐state transformations appear to proceed with barriers (E_a_) that are not reduced and may in fact be substantially higher than those measured for their solution‐phase counterparts. This behavior has been illustrated most clearly for the Diels–Alder cycloaddition, for which activation barriers of approximately 25–29 kcal mol^−1^ have been reported in the solid state, compared with about 15 kcal mol^−1^ for the corresponding solution‐phase reaction, a trend consistent with independent observations by Ma et al. [58]. and Andersen et al. [59]. Collectively, these studies suggest that the mechanochemical environment does not primarily accelerate reactivity by lowering intrinsic activation barriers; rather, its influence operates through repeated generation of productive reactive encounters, stabilization of kinetically trapped nonequilibrium states, and continuous interfacial renewal, mechanisms that are either absent or fundamentally suppressed in solution‐phase chemistry.

This perspective reframes LAG as more than a lubricant. If the solid state does not provide the transition‐state stabilization available in solution, trace liquids may act simultaneously as rheological modifiers, interfacial mediators, and local stabilizing media. Predictive models must therefore integrate impact dynamics, aging, local thermal excursions, and the thermodynamics of solvent‐minimized environments, especially when comparing milling, TSE, RAM, and different reaction classes (Table 1).

**TABLE 1:** Selected conceptual frameworks used to rationalize mechanochemical reactivity are highlighted, along with their interpretive value and present limitations.

**Model/Framework**	**Central Premise**	**Value**	**Limitation**
Empirical descriptions	Correlates outcome with frequency, time, composition, and scale	Ensures practical reporting and reproducibility under matched conditions.	Describes performance but does not explain the origin of reactivity.
Hot‐Spot theory [42]	Attributes reactivity to localized transient heating during impact.	Offers a simple thermodynamic rationale for solid‐state reactivity.	Does not capture the full complexity of modern in situ studies.
Energy budgeting (Jafter et al.) [49]	Reaction occurs once cumulative impact energy exceeds a threshold	Enables comparison across mill types and informs scale‐up analysis.	Neglects dissipative losses and underestimates LAG, ageing, and process history.
Kinematic/Impact‐Driven (Templ et al.) [55]	Prioritizes energy delivered per collision over frequency alone.	Explains why milder, longer protocols can outperform harsher conditions.	Requires more detailed treatment of impact dynamics and material evolution.
LAG/Temperature sensitivity model (Spula et al.) [56]	Treat liquid additives and temperature as key modulators of solid‐state kinetics.	Highlight optimal LAG windows and pronounced thermal sensitivity.	Broader generality remains insufficiently established.

### 1.4 Scope and Structure of This Review

**FIGURE 3:**
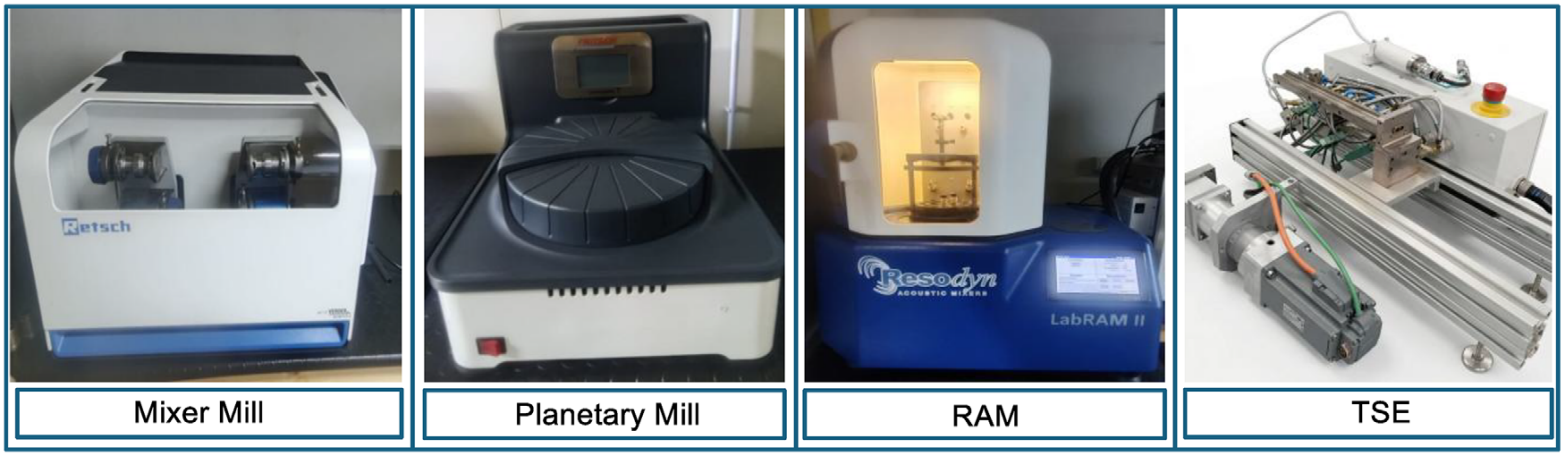
Representative devices used in modern mechanochemical synthesis include automated milling and scalable, continuous processing platforms.

This Review examines the use of mechanical activation to construct metal complexes in the solid state and deploy them, either preformed or generated in situ, in catalytic organic synthesis. The discussion is organized by metal center and ligand class, with emphasis on cases where milling alters reactivity, selectivity, coordination chemistry, reaction time, or scale‐up (Figure 3). At the same time, this Review does not treat these advances uncritically. We examine the limitations that still impede broader uptake, including persistent concerns about reproducibility, the ongoing lack of standardized reporting practices, and the need for a deeper mechanistic foundation that can move the field beyond empirical optimization and toward predictive control. Where relevant, we situate these issues within the broader emergence of in situ analytical tools and process‐aware modes of thinking [36].

## 2 Mechanochemical Generation and Deployment of Metal Catalysts

Mechanochemical processing circumvents key constraints of solution‐phase synthesis by promoting metal–ligand bond formation in an energy‐dense, dynamically evolving medium with minimal solvent and auxiliary input. In this environment, metal species can be generated, activated, and channelled directly into catalytic turnover, allowing complex assembly and catalysis to be unified in a single vessel while minimizing isolation, transfer, and purification—a key feature observed only in a limited number of complexation protocols in solution [60].

### 2.1 First‐Row Metal Catalysis Under Mechanical Activation

First‐row metals offer an attractive sustainability counterpoint to 4d/5d catalysis, but their greater propensity for SET pathways and labile metal–substrate interactions often complicate speciation, selectivity, and structure–reactivity relationships [61, 62, 63].

Mechanochemistry is attractive in this context because solvent‐minimized, high‐contact environments can accelerate elementary steps, displace equilibria, and engage reactive 3d‐metal assemblies that are too transient or weakly populated in solution.

#### 2.1.1 Manganese Mechanocatalysis: C‒H Activation Turned Green

Manganese catalysis offers an attractive combination of abundance, redox flexibility, and catalytic versatility [64]. Its appeal derives from a rare combination of earth abundance and broad catalytic versatility, encompassing C–H functionalization [65, 66, 67], hydrolyzation [68, 69, 70], and hydrogenation chemistry [71, 72]. However, despite this promise, many manganese‐catalyzed protocols still rely on rigorously anhydrous and oxygen‐free conditions, problematic solvent systems, and only moderate reaction rates, which compromise operational simplicity and diminish their overall sustainability profile. Although the use of greener solvents can partly alleviate these concerns, it rarely addresses the intrinsic limitations of the catalytic platform itself, as illustrated by the acid‐mediated alkenylation of indoles reported by Li and coworkers [73].

**SCHEME 1:**
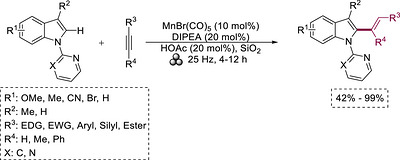
Mechanochemical manganese‐catalyzed regioselective C2 alkenylation of *N*‐aryl indoles, adapted from Ref. [74].

Banerjee and coworkers disclosed a mechanochemical Mn‐catalyzed C2 alkenylation of N‐aryl indoles with alkynes using MnBr(CO)_5_, SiO_2_, and room‐temperature solvent‐free milling for 4–12 h (Scheme 1) [74]. This early low‐valent Mn‐carbonyl protocol avoided external inert‐atmosphere handling, although volatile alkynes such as 2‐butyne and trimethylsilylacetylene remained problematic, likely because vapor‐phase persistence limited contact with the solid mixture [73].

**SCHEME 2:**
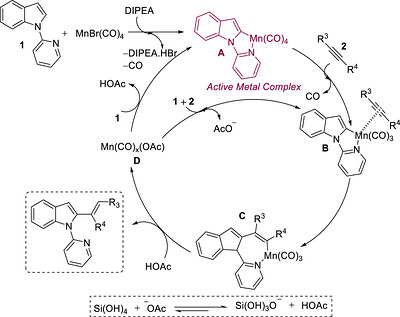
Proposed catalytic pathway for the mechanochemical generation of active manganese intermediate A in the C2 alkenylation of *N*‐aryl indoles, adapted from Ref. [74].

The proposed catalytic cycle begins with base‐assisted generation of a reactive manganese species, followed by heteroaryl‐directed cyclomanganation to give intermediate **A** [73]. Subsequent alkyne insertion furnishes a seven‐membered manganacyclic intermediate, which then undergoes product‐forming protodemetallation to deliver the C2‐alkenylated indole while regenerating the active catalyst (Scheme 2). Mechanistic studies further indicated that electron‐rich indoles react more rapidly, consistent with cyclomanganation and the formation of intermediate **A** as the rate‐determining step. SiO_2_ likely contributes more than dispersion: its silanol‐rich surface may assist proton transfer while improving mixing, consistent with the marked loss of reactivity observed in its absence and with the acid–base behaviour of silica surfaces [75].

Compared with the solution protocol, the mechanochemical variant proceeds at room temperature without bulk solvent or external inert‐atmosphere handling, while maintaining productive catalytic turnover [73]. The enhanced reactivity observed under milling conditions is consistent with continuous surface renewal, which may suppress catalyst passivation and help preserve the catalytic activity throughout the transformation. The improved performance is particularly notable for electron‐poor alkynes, substrates that often prove less competent under solution‐phase conditions [76].

#### 2.1.2 Iron Mechanocatalysis: Reconciling Radical Reactivity With Stereocontrol

Iron catalysis combines abundance, low toxicity, accessible redox chemistry, and broad utility in transfer hydrogenation (TH) [77], borrowing‐hydrogen chemistry [78, 79], oxidation [80, 81], C–C coupling [82], and C–H activation [83]. However, iron catalysis remains intrinsically difficult to control, largely because its propensity for single‐electron transfer (SET) and manifold redox pathways can blur the boundary between productive catalysis and uncontrolled radical reactivity.

**SCHEME 3:**
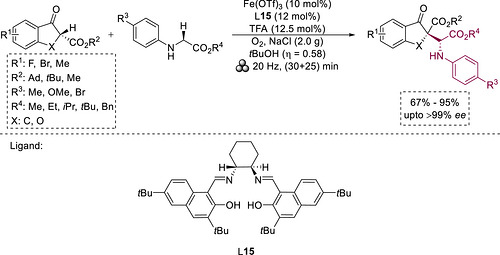
Mechanochemical in situ generated iron complex for C(sp^3^)‒C(sp^3^) dehydrogenative coupling between esters and glycine derivatives, as reported in Ref. [85].

Katayev and coworkers provided a defining early illustration of this reactivity under mechanochemical conditions through an FeCl_3_‐mediated radical ligand‐transfer (RLT) dihalogenation of alkenes, a protocol that privileged operational simplicity and high regioselectivity over short reaction times [84]. Building on this conceptual foundation, Yu and coworkers subsequently disclosed a particularly compelling mechanochemical platform for the stereoselective C(sp^3^)–C(sp^3^) dehydrogenative coupling of esters with glycine derivatives (Scheme 3) [85].

Using an in situ generated iron–salen complex under an O_2_ atmosphere, the authors obtained coupled products with outstanding levels of stereocontrol, reaching up to 99% *ee* and 20:1 *dr*. The decisive parameter was *t*BuOH as LAG additive (*η* = 0.58 µL mg−1), which created a microenvironment capable of stabilising the iminium intermediate generated by acid‐assisted aerobic oxidation of the glycine ester and suppressing racemisation, explaining the sharp improvement in yield and selectivity over both solution‐phase conditions and neat milling; higher LAG loadings were detrimental. Mechanistic experiments established both the radical character of the transformation and the exclusive role of O_2_ as the oxidant. Complementary DFT analysis further indicated that mechanical activation is essential for accessing the catalytically competent L‐Fe‐butanol species, thereby rationalising the difference in the transformation under solution and milling conditions.

**SCHEME 4:**
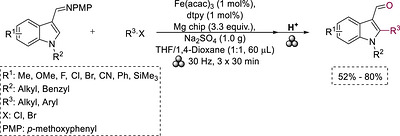
Mechanochemical in situ‐generated iron complex for the C2 alkylation of indoles using in situ‐generated Grignard reagents, as reported in Ref. [86].

In a subsequent study, the same group addressed the longstanding limitations of air‐sensitive organometallic reagents by developing a one‐pot, Fe‐catalyzed mechanochemical C2 alkylation of indoles, enabled by the in situ generation of Grignard reagents (Scheme 4). The protocol proved substantially more sustainable than its solution‐phase counterpart, as evidenced by a reduction in the E‐factor from 221.5 to 46.7 under ball‐milling conditions [86].

##### 2.1.2.1 Preformed Iron Complexes in Mechanochemical Hydroxylation and Wacker‐Type Oxidation

**SCHEME 5:**
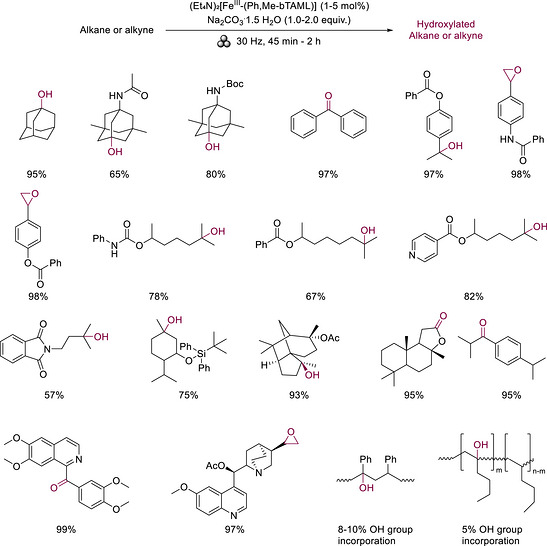
Hydroxylation of unreactive alkanes and alkenes as reported in Ref. [87].

The use of preformed, commercially available iron complexes represents an important frontier in mechanochemical catalysis. Against this background, Gupta and coworkers reported an intriguing iron‐catalyzed platform for the chemoselective hydroxylation of otherwise unactivated C─H bonds using sodium percarbonate (Na_2_CO_3_·1.5 H_2_O_2_) as the oxidant [87]. A modified Fe‐bTAML complex, (Et_4_N)_2_[Fe^III^(Ph,Me‐bTAML)], proved essential because its unusual robustness at high iron oxidation states suppressed catalyst degradation under oxidative milling conditions. Notably, the method operated across a remarkably diverse substrate space, including long‐chain hydrocarbons, poorly soluble polymers such as polystyrene and poly(1‐hexene), and natural products such as ambroxide, all without detectable disruption of the polymer backbone (Scheme 5). Unlike solution‐phase counterparts, which suffer from solvent oxidation, byproduct formation, and poor performance with poorly soluble substrates, the mechanochemical protocol suppresses these pathways and improves yields [88, 89, 90].

**SCHEME 6:**
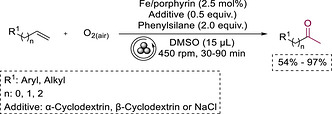
Wacker oxidation conducted under mechanochemical conditions as reported in Ref. [91].

Yu and coworkers further advanced iron mechanocatalysis with a selective Markovnikov Wacker‐type oxidation of aliphatic olefins using an Fe^II^‐porphyrin catalyst, PhSiH_3_ as reductant, and O_2_ to generate the Fe^III^‐alkylperoxy intermediate responsible for ketone formation [91]. Grinding increased the turnover frequency to 77.6 h^−1^, placing the protocol among the most efficient iron‐catalyzed Wacker‐type oxidations of aliphatic olefins (Scheme 6). TEMPO experiments supported a radical pathway, while cyclodextrin was proposed to modulate silane interaction with the Fe^III^‐alkyl intermediate and suppress alkane formation. Together, these examples show that milling can stabilize radical and high‐valent iron manifolds while suppressing solvent‐derived side reactions.

#### 2.1.3 Advances in Mechanochemical Cobalt Catalysis: From Complexation to Piezo‐Redox Systems

Cobalt complexes have emerged as versatile platforms in modern organic synthesis, enabling transformations as diverse as hydroboration [92], hydrosilylation [93], C‒C coupling [94], C‒H activation [95], and cyclization reactions [96].

Cobalt mechanochemistry has developed along two main trajectories: ligand–metal complexation and catalytic organic transformations. Bolm and coworkers first reported the mechanochemical synthesis of [Cp*Co(CO)I_2_] and its use in C2 amidation of indoles [97].

**SCHEME 7:**
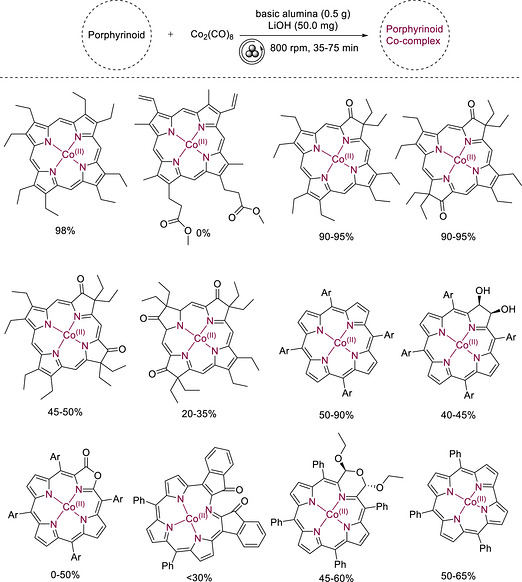
Mechanochemical preparation of Co‐complexes from natural and synthetic porphyrinoids as reported in Ref. [98].

**SCHEME 8:**
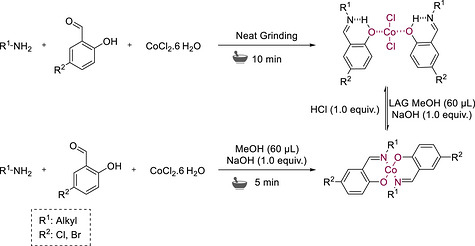
Mechanochemical synthesis of Co–salen complexes by mortar‐and‐pestle grinding, as reported in Ref. [100].

Brückner and coworkers later examined Co_2_(CO)_8_ insertion into natural and synthetic porphyrinoids, revealing both the promise and limits of mechanochemical cobalt complexation, as porpholactones remained unreactive under the tested conditions (Scheme 7) [98]. Zuo et al. [99]. and Chen et al. [100]. independently showed that cobalt–salen complexes can be obtained by mortar‐and‐pestle grinding, including systems capable of reversible monodentate/bidentate switching upon grinding with NaOH or HCl (Scheme 8).

**SCHEME 9:**
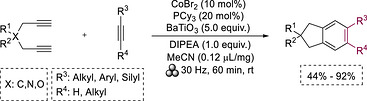
Mechanochemical in situ generated cobalt complex for piezo‐mediated [2+2+2] cycloaddition as reported in Ref. [102].

Within the domain of cobalt‐catalyzed organic transformations, a distinct mechanochemical paradigm has emerged, which exploits piezoelectric materials to access redox reactivity during milling. This emerging mechano‐redox manifold, first pioneered by Ito and coworkers, remains one of the most conceptually distinctive directions in contemporary mechanochemistry [101]. In a recent advance, the same group reported a remarkable piezo‐mediated, cobalt‐catalyzed [2+2+2] cycloaddition between diynes and phenylacetylene derivatives (Scheme 9) [102]. With BaTiO_3_ as a mechanically activated piezoelectric mediator, the milling protocol delivered 96% yield within 60 min, compared with less than 1% in solution, while eliminating sacrificial reductants such as Zn or Mn.

#### 2.1.4 Nickel Mechanocatalysis: Scope, Reactivity, and Process Advantage

Nickel is the most extensively explored first‐row metal in mechanochemical catalysis, combining facile oxidative addition, comparatively slow β‐hydride elimination, and accessible SET pathways [103, 104] across C–C, C–N, hydrogenation, oligomerization, oxidation, and carboxylation manifolds [105, 106, 107, 108, 109, 110, 111, 112, 113, 114].

##### 2.1.4.1 Mechanochemical Synthesis of Nickel Complexes

**SCHEME 10:**
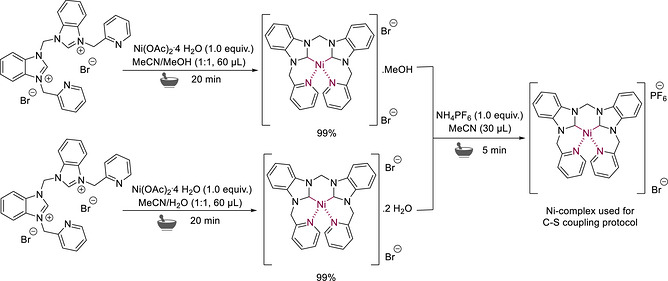
Synthesis of Ni‐complexes having NHC ligands by using a mortar and pestle approach, as reported in Ref. [115].

Guo and coworkers showed that NHC–Ni complexes can be assembled mechanochemically from N‐pyridylmethylbenzimidazole‐derived ligands, affording structurally distinct Ni species that were catalytically competent in C–S coupling (Scheme 10). Under mortar‐and‐pestle conditions, the selected precatalyst delivered the thioether in 93% yield, compared with 25% in solution [115].

**SCHEME 11:**
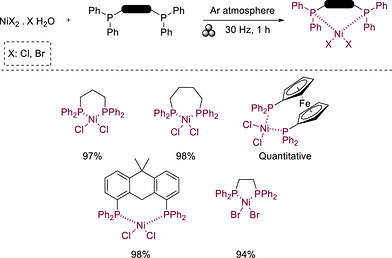
Mechanochemical synthesis of Ni(II)–phosphine complexes, adapted from Ref. [116].

Frąckowiak and Kownacki prepared Ni^II–^phosphine complexes by milling NiCl_2_·6H_2_O with dppe under solvent‐free conditions (Scheme 11) [116]. ^31^P NMR confirmed complex formation, although residual free ligand and phosphine oxide signals indicated partial oxidation during milling and workup. The resulting complexes were then evaluated in cross‐coupling reactions.

##### 2.1.4.2 Cross‐Electrophile Coupling (XEC)

**SCHEME 12:**
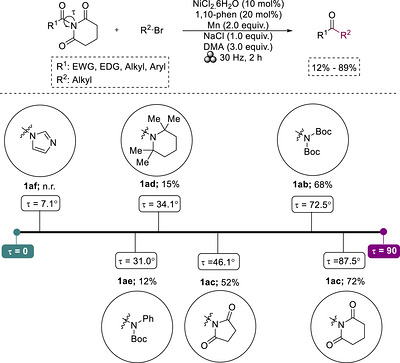
In situ generation of the active nickel complex in mechanochemical cross‐electrophile coupling (XEC), adapted from Ref. [117].

Browne and coworkers introduced a mechanochemical cross‐electrophile coupling (XEC) platform that addressed a key limitation of conventional XEC: the sluggish heterogeneous activation of Zn or Mn reductants under elevated temperatures and long reaction times [117, 118]. Milling NiCl_2_·6H_2_O, 1,10‐phenanthroline, granular Zn, and DMA as LAG additive promoted continuous zinc‐surface renewal and enabled efficient coupling under ambient conditions (Scheme 12).

Building on earlier solution‐phase studies [119, 120, 121], the authors subsequently disclosed a mechanochemical XEC protocol for the synthesis of acyl and heteroaryl products from alkyl halides and so‐called “twisted” amides [117]. Using *N*‐acyl glutarimides as electrophilic partners, the authors established a direct correlation between the catalytic reactivity and the torsional twist angle (*τ*) of the amide N─C(O) bond. Highly twisted amides (*τ* = 87.5°) furnished ketones in excellent yields, whereas nearly planar amides (*τ* ≈ 7°) remained unreactive, underscoring the decisive role of amide geometry in the success of mechanochemical XEC. These studies also resolved a key “reductant problem”: under milling, granular zinc outperformed mossy zinc, flakes, and wire, consistent with more effective surface renewal and sustained metal activation. In a complementary study, Haley and coworkers further clarified the role of DMA as a LAG additive, showing that it is crucial for efficient formation of the XEC products [122].

More recently, Zhu et al. showed that mechanochemical XEC can proceed in as little as 5 min, with markedly higher reactivity than photocatalytic, electrocatalytic, and thermocatalytic alternatives [123].

##### 2.1.4.3 C─S Bond Formation and Alkene Difunctionalization

The sulfenylation of aryl halides remains a cornerstone of pharmaceutical and natural product synthesis, providing direct access to biologically important thioethers [124, 125]. Mechanochemical C–S coupling was first achieved using a Pd‐PEPPSI catalyst [126]. However, nickel‐catalyzed variants of these transformations were subsequently pioneered by Guo and coworkers using aryl iodides and aromatic sulfur surrogates such as mercaptobenzothiazoles and disulfides [127]. A complementary protocol based on aryl disulfides (ArS–SAr) was also developed, in which I_2_ was employed to enhance the electrophilicity of the sulfur source. Single‐crystal X‐ray analysis of the thioether products confirmed structural parameters fully consistent with literature precedents [128]. Interestingly, unlike BHT, TEMPO did not function merely as a radical trap; instead, it completely suppressed catalysis, most likely through chelation to the active nickel center [129]. Competitive experiments further revealed that electron‐deficient aryl iodides exhibit enhanced reactivity, consistent with a Ni(0)/Ni(II) catalytic manifold.

**SCHEME 13:**
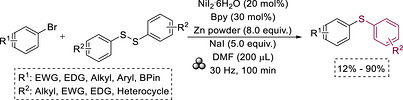
In situ generation of the active nickel complex in mechanochemical C─S bond formation, adapted from Ref. [130].

The practical viability of mechanochemical Ni‐catalyzed C─S bond formation was reinforced by subsequent studies from the same group (Scheme 13) [130].

**SCHEME 14:**
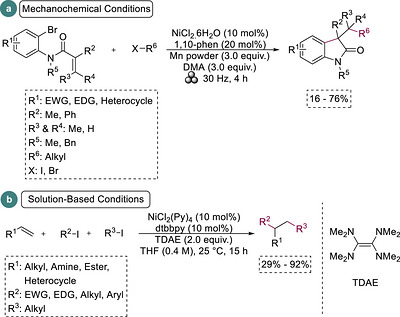
In situ generated Ni‐complex in (a) mechanochemical intramolecular alkene dicarbofunctionalization (DCF) [135], (b) solution‐based alkene dicarbofunctionalization as reported in Ref. [134].

Reductive dicarbofunctionalization of alkenes provides a powerful entry to cyclized, pharmaceutically relevant scaffolds such as 3,3‐disubstituted oxindoles and indolines [131, 132, 133]. To overcome the elevated temperatures, inert‐atmosphere handling, prolonged reaction times, and air‐sensitive reductants such as TDAE that typically burden solution‐phase protocols [134], Browne and coworkers developed a robust mechanochemical nickel‐mediated strategy for the intramolecular dicarbofunctionalization of alkenes between iodoalkanes and fluoro‐substituted aryl acrylamides (Scheme 14) [135].

Using a NiCl_2_·6H_2_O/1,10‐phenanthroline catalyst system together with powdered Mn as the reductant, the authors obtained the cyclized products in excellent yields, along with only minor amounts of the competing XEC product. The protocol proved particularly effective for sterically encumbered substrates, including *ortho*‐methyl‐substituted oxindoles, which are often difficult to access under solution‐phase conditions. Notably, preliminary enantio‐induction studies using a chiral PyrOx ligand already yielded the product with 46% *ee*, suggesting future opportunities for asymmetric development.

The authors proposed two competing scenarios: a SET pathway involving a transient alkyl radical, and an organomanganese pathway in which in situ‐generated organomanganese intermediates add across the alkene before converging on the same products, reported via an organozinc analogue [136]. The observation that the reaction also proceeds with aryl bromides, substrates generally less prone than aryl iodides to engage in radical generation, points to a potentially dominant role for the organomanganese pathway under the high‐energy conditions of ball milling. In this broader context, complementary nickel‐catalyzed dicarbofunctionalization protocols employing Zn as sacrificial reductant were subsequently reported by Fan et al. [137]. and Lei et al. [138].

##### 2.1.4.4 Decarbonylative Coupling and Defluorinative Functionalization

**SCHEME 15:**
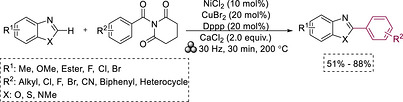
In situ generation of the active nickel complex in mechanochemical decarbonylative heteroarylation, adapted from Ref. [139].

Szostak and coworkers further extended the scope of mechanochemical nickel‐catalyzed reactions by developing a decarbonylative heteroarylation platform (Scheme 15) [139]. Using a dual NiCl_2_/CuBr_2_/dppp catalytic system, the authors enabled concurrent activation of the amide C(acyl)─N bond and the heteroarene C─H bond.

By employing CaCl_2_ as a desiccant auxiliary and conducting the reaction at 200°C in a PID‐controlled milling setup (PID = proportional–integral–derivative), the authors achieved high chemoselectivity in decarbonylative C(sp^2^)–C(sp^2^) coupling. This transformation remains difficult to achieve with comparable selectivity under conventional thermally driven solution‐phase conditions [140, 141]. Notably, the thermal milling setup combines precise temperature regulation (±1°C) with favorable energy efficiency, operating at 400 W compared with 2000 W for a heat gun and 650 W for a conventional stirrer. An impact‐dependent comparison using milling balls of different densities further showed that stainless‐steel media outperformed zirconia, with the latter leading to markedly diminished product formation.

In further advances in nickel mechanocatalysis, Iaroshenko and coworkers demonstrated a nickel‐catalyzed selective defluorinative coupling of trifluoroacetamides, providing access to arylated amides through four distinct classes of coupling partner: arylboronic acids, trimethoxyphenylsilanes, diaryliodonium salts, and dimethylphenylsulfonium salts [142].

**SCHEME 16:**
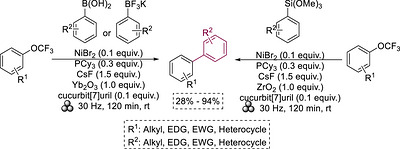
Mechanochemical in situ generated nickel complex in defluorinative coupling of trifluoroacetamides as reported in Ref. [143].

Under mechanochemical conditions, selective cleavage of the amide‐associated N(O = C)─CF_3_ bond could be achieved while leaving other fluorinated motifs, including CF_3_─O, Ar─CF_3_, and Ar─F bonds, intact. The same group subsequently showed that trifluoromethoxyarenes can serve as halide surrogates in a mechanochemical nickel‐catalyzed cross‐coupling platform (Scheme 16) [143].

Previous attempts to exploit trifluoromethoxyarenes in cross‐coupling have relied largely on Grignard chemistry under solution‐phase conditions. For example, Dankwardt obtained only 30% yield of the coupled product after 15 h at 80°C [144], whereas Xie et al. achieved up to 83% yield only under substantially more forcing conditions (120°C, 16 h) [145]. Mechanochemical activation of C─OCF_3_ bonds, therefore, provides a milder entry to this cross‐coupling manifold.

##### 2.1.4.5 Direct Deployment of Nickel Complexes in Mechanochemistry

**SCHEME 17:**
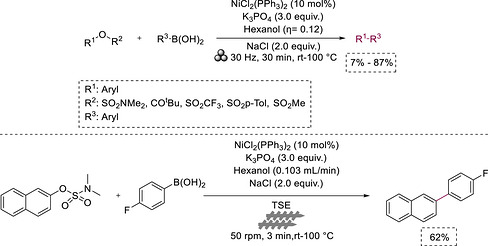
Mechanochemical Suzuki–Miyaura coupling catalyzed by nickel, as reported in Ref. [147].

Leitch and Browne demonstrated nickel catalysis in the solid state through a mechanochemical Suzuki–Miyaura coupling of aryl sulfamates and activated phenols [146, 147]. Optimization of filling degree, jar geometry, and temperature enabled efficient coupling, with a thermostated band heater providing reproducible PID‐controlled heat transfer (Scheme 17). The protocol was also translated to TSE, achieving a throughput of 5.35 g h^−1^ and a space–time yield of 3.6 × 10^3^ kg m^−3^ day^−1^.

**SCHEME 18:**
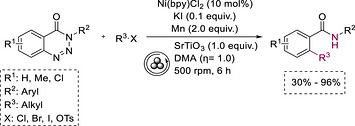
Mechanochemical functionalisation of benzotriazinones, adapted from Ref. [148].

The synthetic reach of nickel‐catalysed coupling was further extended through a manganese‐assisted, denitrogenative ring‐opening of benzotriazinones [148]. In this transformation, ring opening enables selective incorporation of the sp^3^ carbon of an alkyl halide or pseudohalide, providing access to a range of *ortho*‐alkylbenzamides (Scheme 18).

This radical‐mediated process is promoted by incorporating the piezoelectric material strontium titanate (SrTiO_3_), which acts in concert with manganese during the single‐electron‐transfer (SET) event that generates the alkyl radical. Once formed, the alkyl radical is intercepted by the nickel catalytic cycle to generate a Ni^III^ intermediate, which then undergoes reductive elimination to furnish the functionalised aromatic product.

**SCHEME 19:**
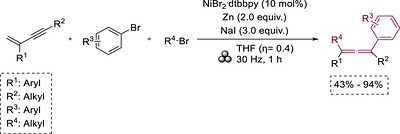
Alkene dicarbofunctionalization through a three‐component reductive protocol as reported in Ref. [150].

Nickel catalysis has also proven central to alkene dicarbofunctionalization, enabling the simultaneous installation of two carbon fragments across an unsaturated bond in a single synthetic step [149]. Wei and coworkers reported a three‐component nickel‐catalysed reductive dicarbofunctionalization of terminal alkenes to allenes using Zn as reductant and NaI as additive (Scheme 19) [150]. The protocol shortened reaction times, lowered reaction temperatures, avoided handling preformed organozinc reagents, and tolerated broad functional groups [151]. TEMPO experiments supported a radical pathway, while zinc regenerated the active nickel species.

**SCHEME 20:**
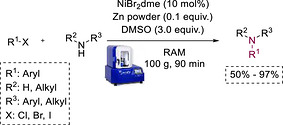
Buchwald coupling under RAM conditions as reported in Ref. [152].

Rueping and coworkers used RAM for nickel‐catalysed Buchwald‐type couplings, combining 96‐well screening with ex situ GC–MS to accelerate optimisation and preserve reproducibility [152]. The method coupled diverse aryl halides with piperidine, morpholine, and anilines, including on a 5 g scale; direct comparison with ball milling showed comparable or superior RAM performance, with yields of 50%–97% versus 32%–96% (Scheme 20).

**SCHEME 21:**
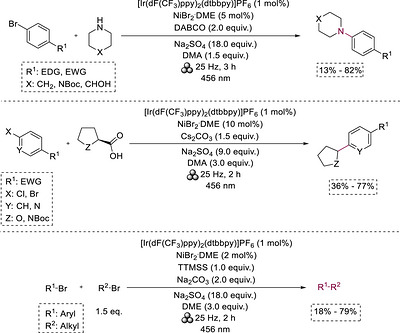
Nickel‐based mechanophotocatalytic cross‐coupling under mechanochemical conditions, as reported in Ref. [153].

Zysman‐Colman and coworkers further expanded the reactivity landscape of nickel catalysis by introducing a photomechanochemical platform for cross‐coupling reactions [153]. Their setup employed polypropylene Eppendorf vials, either within a PMMA milling jar or in a dedicated holder within a mechano‐photocatalytic reactor, thereby enabling simultaneous mechanical agitation and light irradiation. Across a range of transformations, including aryl amination, decarboxylative and deborylative cross‐coupling, and XAT‐enabled processes, the authors showed that reactions performed under aerobic mechanochemical conditions matched, and in some cases outperformed, their strictly anaerobic solution‐phase counterparts (Scheme 21).

These protocols were unified by a common reaction design, typically employing Na_2_SO_4_ as a grinding auxiliary together with only a minimal liquid additive, such as DMA or DME. Photoluminescence studies provided a compelling rationale for the enhanced reactivity, revealing that confining the reagents in the solid state markedly suppresses oxygen‐induced quenching compared with solution‐phase conditions.

Overall, nickel mechanocatalysis now spans heated milling, RAM, photomechanochemistry, and the direct use of milling media as catalyst sources.

#### 2.1.5 Mechanochemical Copper Catalysis: From NHC‐Complex Synthesis to Mechano‐Redox Transformations

**SCHEME 22:**
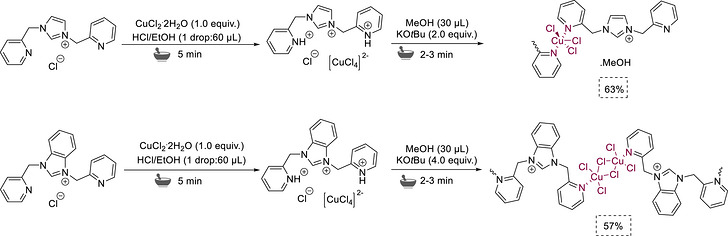
Synthesis of NHC copper complexes by using a mortar and pestle approach, as reported in Ref. [160].

Copper mechanochemistry benefits from the accessible Cu^I^/Cu^II^ redox couple and the geometric flexibility of copper complexes, which support oxidation, cycloaddition, cross‐coupling, and heterocycle‐forming manifolds [154, 155, 156, 157, 158]. NHCs stabilize copper in multiple oxidation states and improve robustness [159]. Mechanochemical dehydrohalogenation of imidazolium or benzimidazolium precursors with copper salts provided polymeric or binuclear NHC–Cu complexes, one of which catalyzed C–S coupling in 99% yield using MeCN as LAG additive (Scheme 22) [160]. Exposure to HCl vapor reversibly regenerated the corresponding metal–organic salt, accompanied by a green‐to‐yellow color change [161].

**SCHEME 23:**
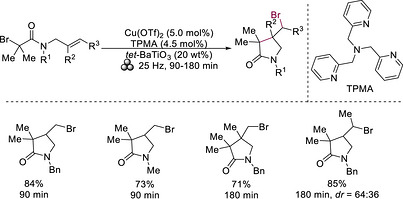
Mechanochemical in situ generated copper complex in atom transfer radical cyclization (ATRC) as reported in Ref. [162].

Bolm and coworkers [162] used a piezo‐mediated mechano‐redox strategy for Cu‐catalyzed ATRC, in which BaTiO_3_ reduces [Cu^II^(TPMA)(OTf)_2_] in situ to catalytically competent Cu^I^ under milling conditions (Scheme 23) [163, 164]. Reactivity depended on the balance between collision frequency and impact intensity: eight 5 mm balls gave 97% yield, whereas one 10 mm ball or thirty‐nine 3 mm balls gave only 32% and 40%, respectively.

The same group further broadened the scope of copper‐catalyzed mechano‐redox chemistry by converting allenes into sulfoximidoyl‐substituted allyl chlorides under milling conditions [165]. Efficient product formation required silica in combination with BaTiO_3_. In a related advance, Zheng and coworkers developed a copper‐catalyzed mechano‐redox platform for forging both C─C and C─N bonds from α‐bromo‐*N*‐sulfonyl amides, thereby granting access to oxindoles and α‐arylacylamides [166]. Control experiments suggested that the key amidyl radicals generated in situ are quenched through protonation, promoted by the LAG additive and/or trace water present in the reaction mixture.

**SCHEME 24:**
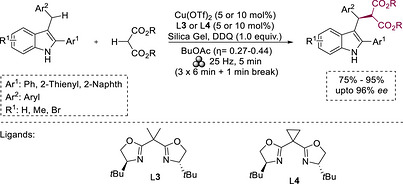
In situ generation of active copper complexes in mechanochemical enantioselective CDC, adapted from Ref. [168].

In a complementary direction, Su and coworkers disclosed a mechanochemical copper‐catalysed cross‐dehydrogenative coupling (CDC) that delivered notable enantiocontrol (Scheme 24) under conditions substantially milder than those required in the corresponding solution‐phase protocol (0°C, 21 h) [167, 168].

In this study, LAG with *n*‐BuOAc proved decisive, increasing stereocontrol from ca. 30% to ca. 50%, underscoring that LAG can serve as a genuine design parameter rather than a passive processing aid. Computational studies further indicated that *n*‐BuOAc promotes the formation of a catalytically competent Cu(II) species, thereby facilitating access to the mechanochemical manifold.

Additional copper‐driven mechanocatalytic couplings have likewise been demonstrated by Zhang et al. in C–H difluoroalkylation [169], by Liu and coworkers in EMM‐promoted C–O coupling en route to diaryl ethers [170], and by Karmakar et al. in a three‐component click protocol employing organoselenides [157].

Particularly striking is the EMM‐promoted C–O coupling platform [170], in which high‐speed rotation of electrically conductive rods within a magnetic field generates polarised free electrons that facilitate the catalytic redox cycle by reducing Cu(II) in situ to catalytically active Cu(I) [171]. More broadly, this inventive use of EMM demonstrates that the design of milling components can itself become a mechanistic variable rather than remain merely an incidental feature of the apparatus.

##### 2.1.5.1 Copper Complexes in Mechanocatalytic Synthesis

**SCHEME 25:**
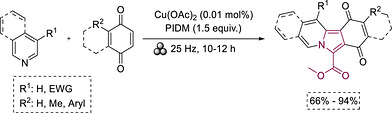
Mechanochemical synthesis of azaheterocyclic scaffolds, adapted from Ref. [172].

Sen reported a Cu(OAc)_2_‐mediated multicomponent mechanochemical strategy involving an azaheterocycle, phenyl iodonium dimethyl malonate (PIDM, a hypervalent iodine reagent), and 1,4‐quinone derivatives to access azaheterocyclic scaffolds (Scheme 25) [172]. Control experiments with TEMPO and BHT excluded radical intermediates and instead supported an ionic pathway, most plausibly a [3+2] cycloaddition between a pyridinium ylide intermediate and the 1,4‐quinone core. This mechanistic interpretation is consistent with the outcome previously observed in the Rh‐catalyzed reaction of pyridine with *o*‐hydroxyphenyliodonium dimethyl malonate [173]. Direct comparison with solution‐based methods showed that the mechanochemical protocol outperformed the corresponding solution‐phase transformation and remained effective even at lower temperatures (up to 90°C less) [174]. It also compared favorably with a related ruthenium‐catalyzed protocol by implementing a more environmentally friendly catalytic center [175].

Subsequent photophysical studies further showed that the benzo[5,6]isoindolo[2,1‐b]isoquinoline derivative, by virtue of its extended π‐system, displays a quantum yield of 12.6% and can serve as a potential intracellular fluorescent probe.

**SCHEME 26:**
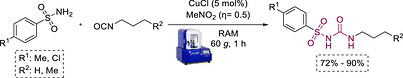
Mechanochemical synthesis of sulfonylureas under RAM conditions, adapted from Ref. [176].

Friščić and coworkers extended copper catalysis to RAM via a CuCl‐mediated coupling of a sulfonamide with an isocyanate, enabling the solid‐state synthesis of the API tolbutamide with nitromethane as the liquid additive (*η* = 0.5 µL mg−^1^) (Scheme 26) [176]. The transformation was scaled to 30 g, affording pure tolbutamide in 86% yield after simple treatment with aqueous Na_2_H_2_EDTA.

#### 2.1.6 Mechanochemical Synthesis of Zinc Complexes

**SCHEME 27:**
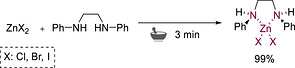
Preparation of Zn‐complexes using *N,N′*‐diphenylethylenediamine, as reported in Ref. [177].

Feng et al. prepared zinc complexes by grinding N,N′‐diphenylethylenediamine with halogenated zinc salts, with PXRD confirming coordination‐compound formation (Scheme 27) [177]. The complexes catalyzed C–S coupling of α‐haloacetophenones with sulfur heterocycles, likely through transient zinc disulfide intermediates.

In a one‐pot cascade, complex synthesis and C–S coupling were integrated without intermediate isolation, streamlining the workflow and revealing a meaningful halide effect on catalytic performance.

**SCHEME 28:**
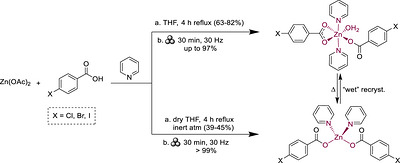
Synthetic routes to zinc complexes in solution and under mechanochemical conditions, adapted from Ref. [178].

The ability of mechanochemistry to deliver zinc complexes bearing nonchelating ligands with high selectivity and reproducibility was further demonstrated by Cagossi et al., who compared mechanochemical and solution‐phase routes to heteroleptic Zn(II) complexes containing pyridine and *p*‐halogenobenzoate ligands (Scheme 28) [178]. Under neat grinding conditions, conversion was achieved within minutes, and PXRD analysis showed that the mechanochemically obtained crystalline phases were structurally identical to those formed in solution. Dry milling provided anhydrous complexes without specialized moisture‐exclusion equipment, an outcome difficult to secure in solution without glovebox or Schlenk‐line techniques. The selectivity of this process was shown not to be accidental: even with nonchelating ligands, which provide a weaker thermodynamic driving force for complex formation than chelating analogues, the reactions proceeded rapidly and cleanly.

**FIGURE 4:**
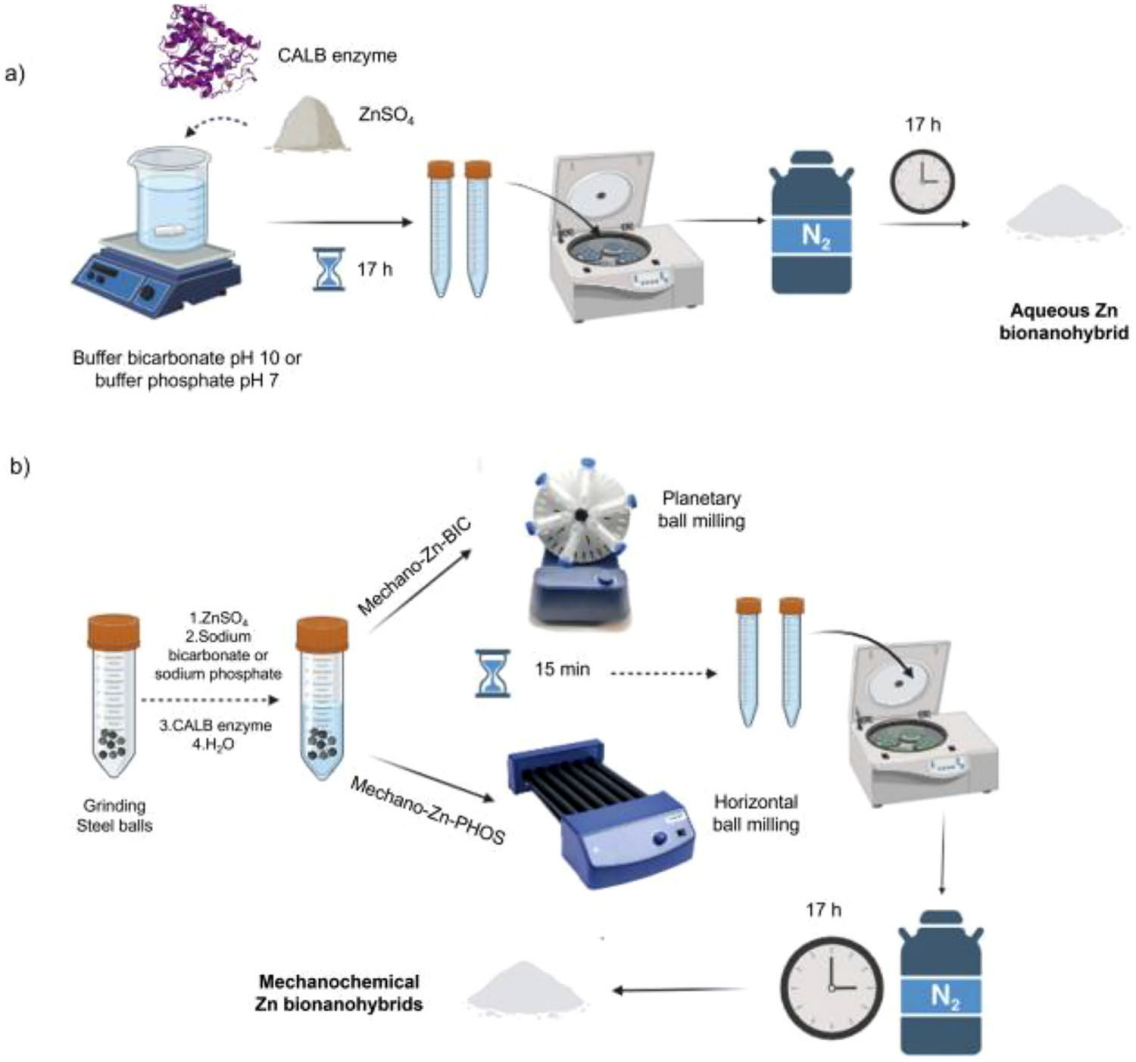
Preparation of Zn‐based bionanohybrids through two distinct approaches: (a) aqueous route, (b) mechanochemical route. Reproduced from Ref. [179].

Mechanochemical zinc chemistry has also been extended to the preparation of novel nanomaterials with enzyme‐mimetic activity. Garcia‐Sanz et al. reported the mechanosynthesis of zinc bionanohybrids by combining *Candida antarctica* lipase B (CALB) with zinc salts and phosphate or bicarbonate salts under ball‐milling conditions [179]. The resulting hybrid nanomaterials, whose properties depended strongly on milling‐ball size, displayed enhanced enzyme‐mimetic activity relative to analogues prepared by conventional aqueous synthesis, an outcome attributed to the formation of smaller, more homogeneous nanostructures under mechanochemical conditions (Figure 4). Reaction times were drastically shortened relative to the solution‐based reference procedure, and synthesis proceeded at room temperature in the near‐absence of solvent.

Zinc mechanochemistry therefore spans C–S coupling, dry heteroleptic complex synthesis, and functional biohybrid nanomaterials.

### 2.2 Later Transition‐Metal Catalysis Under Mechanical Activation

Second‐ and third‐row metal complexes remain central to homogeneous catalysis because their orbital energetics and stronger metal–ligand interactions favour robust two‐electron manifolds [180]. The question here is whether mechanochemistry offers these systems advantages beyond solvent minimization, including altered selectivity, distinctive reactivity, or operational simplification [181, 182].

#### 2.2.1 Molybdenum Complexes in Mechanochemical Nitrogen Fixation

**SCHEME 29:**
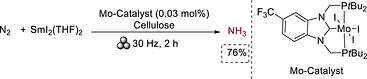
Mechanochemical nitrogen fixation using a molybdenum PCP‐type pincer complex, adapted from Ref. [186].

Molybdenum occupies a defining role in nitrogen fixation, both in biological catalysis and synthetic small‐molecule activation [183]. Recent advances have shown that nitrogen fixation can also be promoted under mechanochemical conditions, opening a conceptually distinct alternative to classical solution‐phase approaches [184, 185]. Nishibayashi and coworkers demonstrated this concept using molybdenum PCP‐type pincer complexes in combination with SmI_2_(THF)_2_ as the reductant and a range of protic additives (Scheme 29) [186].

Diffuse‐reflectance UV/Vis spectroscopy and X‐ray studies suggested that slow cellulose–SmI_2_(THF)_2_ coordination delays formation of catalytically competent species during milling, accompanied by oxidation of Sm^II^ to Sm^III^.

Related PCP‐type Mo complexes bearing trifluoromethyl‐substituted pincer ligands and pentaerythritol as proton donor also furnished ammonia. The NH_3_ remained trapped in the solid matrix, probably through coordination to in situ‐formed Sm^III^, and was released by milling with base. NMR, EI‐TOF MS, and PCET model studies supported a molybdenum nitride intermediate en route to ammonia.

#### 2.2.2 Ruthenium Mechanocatalysis: From Asymmetric Synthesis to Distal Functionalization

**SCHEME 30:**
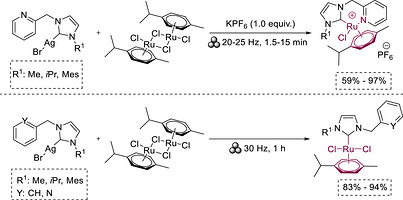
Mechanochemical synthesis of NHC–Ru complexes, adapted from Ref. [193].

Ruthenium mechanocatalysis illustrates how milling can go beyond solvent minimization in TH, borrowing‐hydrogen chemistry, C–H functionalization [187, 188, 189], and metathesis [190, 191]. In this context, ruthenium mechanocatalysis has evolved into one of the clearest test cases for determining when, and why, mechanochemistry offers advantages that extend beyond simple solvent minimization in later transition‐metal catalysis [192]. In a representative example, Bantreil and coworkers developed a mechanochemical route to Noels‐type ruthenium precatalysts, a family widely used in metalation chemistry and in the ring‐opening metathesis polymerization (ROMP) of norbornene derivatives [193]. Their workflow proceeds through *N*‐alkyl imidazolium salts, which are metalated by grinding with Ag_2_O to generate the corresponding Ag–NHC intermediates, followed by milling with [Ru(*p*‐cymene)Cl_2_]_2_ for 1 h to furnish the targeted Ru complexes (Scheme 30).

Interestingly, pyridine‐containing substrates did not chelate under the standard conditions. Chelation could, however, be induced by the addition of KPF_6_ together with the use of a PTFE jar and a lower milling frequency, plausibly because PF_6_−‐containing conditions can weaken the halide coordination to the metal center and thereby favour the new mode of metal‐ligand interaction [194]. Among the systems prepared, the mesitylene‐derived complex displayed the highest ROMP activity, affording poly(norbornene) in 99% yield within 40 min [193].

**SCHEME 31:**
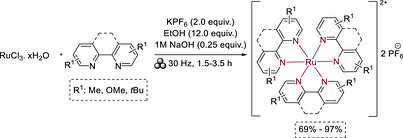
Mechanochemical synthesis of Ru polypyridyl complexes, adapted from Ref. [195].

The target complex was prepared conveniently by milling RuCl_3_, KPF_6_, and 2,2′‐bipyridine in the presence of ethanol and aqueous NaOH as liquid additives, thus avoiding the need for conventional bulk solvents (Scheme 31). To enable irradiation during milling, transparent epoxy‐resin jars were employed, allowing efficient coupling of mechanical agitation with blue‐LED illumination. This photomechanochemical setup enabled reductive dehalogenation of α‐haloesters using the preformed Ru catalyst in combination with Hantzsch amide and DIPEA, with DMAc serving as the liquid additive [195].

**SCHEME 32:**
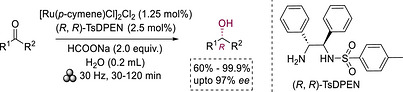
In situ generation of the active Ru complex in mechanochemical ATH of ketones, adapted from Ref. [196].

Asymmetric transfer hydrogenation (ATH) of ketones remains one of the most valuable transformations. Against this backdrop, Szőllősi and coworkers developed a high‐performance ruthenium‐catalyzed mechanochemical ATH of acetophenone to 1‐phenylethanol using a [RuCl_2_(*p*‐cymene)]_2_/TsDPEN system with HCOONa as reductant (Scheme 32) [196]. The mechanochemical protocol displayed striking kinetic superiority over its solution‐phase counterpart, delivering a TOF of 139.2 h^−1^ versus 28.8 h^−1^ in solution while maintaining excellent enantioselectivity of up to 97% *ee*. Its advantage became even more pronounced in the reduction of 6‐methoxytetralone, for which the batch process gave less than 1% conversion, whereas ball milling afforded 97% conversion and 94% *ee*.

**SCHEME 33:**
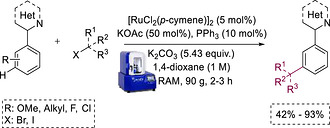
RAM‐mediated in situ generation of the active Ru complex for *meta*‐C–H alkylation of 2‐phenylpyridines with alkyl halides, adapted from Ref. [197].

The reach of ruthenium mechanocatalysis was further expanded by a landmark study from Rueping and coworkers, who investigated Ru‐catalyzed meta‐C–H alkylation of 2‐phenylpyridines with alkyl halides under RAM conditions (Scheme 33) [197]. Using a [RuCl_2_(*p*‐cymene)]_2_/PPh_3_ system under 90 *g* of vertical acceleration for 2–3 h, the authors obtained the *meta*‐alkylated products in excellent yields. Particularly noteworthy is that this mechanochemical protocol proved effective for distal *meta*‐C–H activation using [RuCl_2_(*p*‐cymene)]_2_ in combination with KOAc and phosphine ligands, thereby extending the observations of Ackermann and coworkers: in solution, the simple combination of [RuCl_2_(*p*‐cymene)]_2_ and phosphine was inactive, whereas the acetate complex [Ru(OAc)_2_(*p*‐cymene)]_2_ displayed reactivity [198].

##### 2.2.2.1 Commercial Ruthenium Complexes in Mechanocatalysis

**SCHEME 34:**
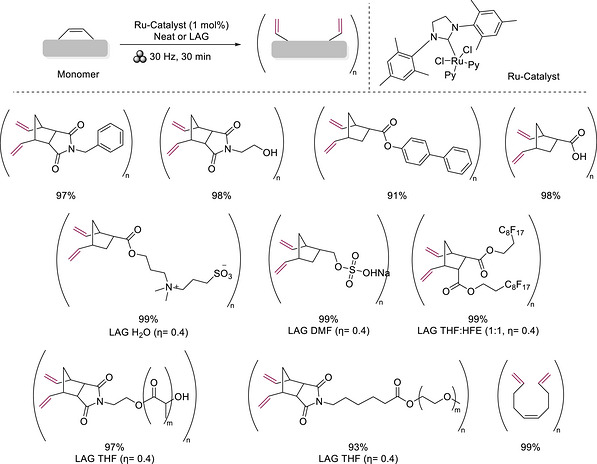
Mechanochemical ROMP using commercial ruthenium catalysts, adapted from Ref. [199].

Lee et al. investigated ROMP under mechanochemical conditions [199]. Under ball‐milling conditions, Ru–alkylidene catalysts enabled access to a diverse polymer family, including materials derived from monomers that are otherwise difficult to process because of their limited solubility (Scheme 34). Control experiments further revealed that LAG plays a dual role in this system: it improves dispersion of the growing polymer phase while simultaneously enhancing overall reaction efficiency, thereby exerting a direct mechanistic influence on the metathesis manifold [200].

Ruthenium catalysts have also proved exceptionally valuable in transfer hydrogenation (TH), where they frequently combine mild operating conditions with high selectivity in asymmetric synthesis [201]. A one‐pot mechanochemical sequence combined asymmetric Michael addition with chiral reduction to furnish enantioenriched δ‐hydroxysulfones [202]. Central to the success of this methodology was the dual use of β‐cyclodextrin (β‐CD) and cetyltrimethylammonium bromide (CTAB). β‐CD effectively compartmentalized the two catalytic events, enabling the initial Michael addition and, upon interaction with CTAB, promoting transfer of the intermediate to the ATH catalyst. This design minimized deleterious cross‐talk between the two chiral catalysts and enhanced overall selectivity. In the Michael addition step, a chiral squaramide operating in the presence of β‐CD delivered a TOF of 39.2 h^−1^, far exceeding that of the corresponding solution‐phase process (4.8 h^−1^).

**SCHEME 35:**
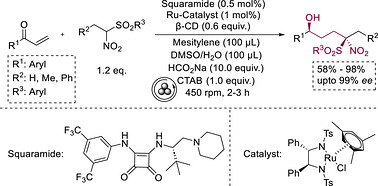
Mechanochemical enantioselective Michael addition/ATH cascade to *δ*‐hydroxysulfones, adapted from Ref. [202].

The subsequent ATH, catalysed by (η^6^‐mesitylene)RuCl(TsDPEN) with HCOONa as hydrogen donor and DMSO/H_2_O as liquid additive, proceeded smoothly to furnish the enantioenriched alcohol. Continued use of β‐CD and CTAB was essential to preserve catalyst compatibility throughout the cascade. The scope encompassed a broad series of enones, which were converted efficiently into the corresponding 1,4‐diastereomeric *δ*‐hydroxysulfones with excellent tolerance toward both electron‐withdrawing and electron‐donating substituents (Scheme 35). The sequence also proved amenable to gram‐scale synthesis.

**SCHEME 36:**
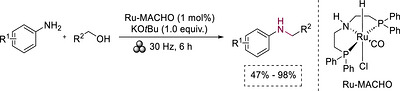
Mechanochemical borrowing‐hydrogen *N*‐alkylation of amines using a commercial ruthenium catalyst, adapted from Ref. [203].

Porcheddu and coworkers reported Ru‐MACHO‐catalysed borrowing‐hydrogen *N*‐alkylation of amines under ball milling, in which alcohol dehydrogenation, imine formation, and hydrogen transfer occur within the same catalytic manifold (Scheme 36) [203]. The system reached TON values above 2000 and converted a broad range of aromatic amines into secondary amines within 6 h, outperforming earlier classical protocols [204].

By contrast, aliphatic amines required more forcing conditions, specifically external heating to 80°C during milling, monitored by IR thermometry, reflecting their intrinsically lower reactivity. Notably, these substrates remained essentially unreactive under analogous solution‐phase conditions.

**SCHEME 37:**
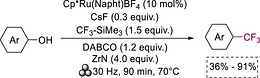
Ruthenium‐catalysed mechanochemical activation of phenols for trifluoromethylation, adapted from Ref. [209].

The synthetic reach of commercial Ru catalysts was further extended to phenol activation, enabling access to diverse valuable functionalities [205, 206, 207, 208]. Iaroshenko and coworkers demonstrated direct trifluoromethylation of phenols through hydroxyl‐group substitution [209]. In this protocol, CF_3_SiMe_3_ served as a trifluoromethylating reagent in combination with the ruthenium catalyst Cp*Ru(Naphthyl)BF_4_, DABCO as a base, CsF to activate the CF_3_ source, and ZrN as an oxyphilic additive. Conducted at 70°C, the reaction furnished a broad range of CF_3_‐substituted arenes (Scheme 37).

Because no reactivity was observed in solution, even under forcing conditions, the authors attributed the success of the mechanochemical protocol to ZrN's poor solubility in conventional organic media and therefore investigated the mechanism using DFT calculations. These studies revealed a strong dependence on ZrN, which promotes a key ligand‐to‐metal charge‐transfer event within the ruthenium sandwich complex. This step facilitates nucleophilic attack by the CF_3_ fragment generated from the activated CF_3_SiMe_3_/CsF system, ultimately leading to the release of silanol (SiMe_3_OH) and the formation of the trifluoromethylated arene. Installation of fluorinated substituents such as F, CF_3_, and OCF_3_ on aryl rings was further expanded through piezoelectric‐material‐assisted ruthenium catalysis, using deamidation and desulfonamidation of aryl primary amides and sulfonamides, respectively [210, 211].

Together, these examples show that commercial Ru catalysts can exploit milling to process poorly soluble substrates, compartmentalise incompatible catalytic events, and access fluorination and trifluoromethylation manifolds that remain difficult under conventional conditions.

#### 2.2.3 Rhodium Complexes in Mechanochemical C–H Methylation and N–H Insertion

Rhodium mechanocatalysis remains less developed than its palladium and ruthenium counterparts, but selected examples show that mechanical input can influence site selectivity, carbene insertion, and transfer‐hydrogenation chemistry [212, 213, 214, 215, 216, 217, 218].

**SCHEME 38:**
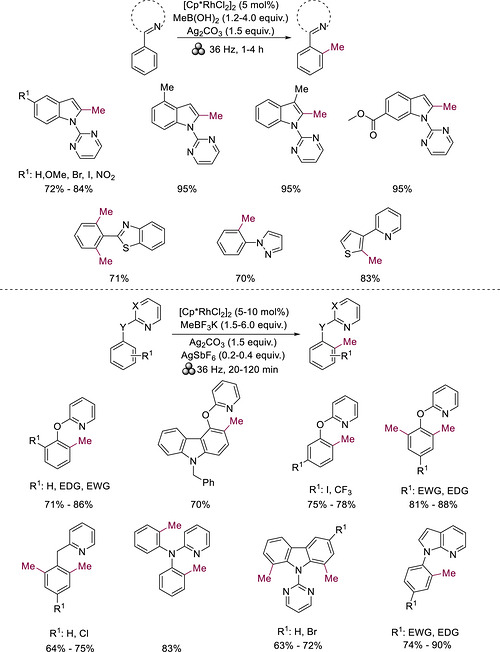
Mechanochemical C–H methylation of azaheterocycles, adapted from Ref. [219].

Mechanochemical C–H methylation was investigated by Pilarski and coworkers, who functionalized azaheterocycles with [Cp*RhCl_2_]_2_ under oxidative conditions [219]. The reaction was proposed to proceed through the formation of either a five‐ or six‐membered rhodacyclic intermediate, each requiring slightly different conditions for efficient turnover. In the latter case, corresponding to the less thermodynamically favoured pathway, successful methylation required the use of MeBF_3_K as methyl source together with a substoichiometric amount of AgSbF_6_ (Scheme 38).

Extension to indoline derivatives revealed milling frequency as a selectivity parameter: 25 Hz favoured C7 methylation, whereas 36 Hz induced dehydrogenation followed by C2 methylation. Subsequent grind‐and‐heat experiments showed that homogenisation alone was insufficient; productive reactivity required softening or melting of the substrate or reaction mixture under the applied thermal regime [220].

**SCHEME 39:**
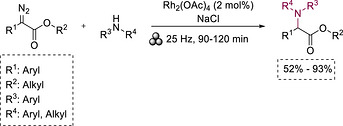
Mechanochemical rhodium‐catalysed synthesis of α‐amino acid derivatives, adapted from Ref. [221].

Bolm and coworkers further extended rhodium mechanocatalysis to carbene N–H insertion reactions of α‐diazo esters for the synthesis of α‐amino acid derivatives [221]. The protocol tolerated diverse functional groups and gave biologically relevant α‐amino acid derivatives, including menthol‐ and borneol‐derived derivatives (Scheme 39). Temperature evolution during milling was monitored, and the mechanochemical process was directly compared with the corresponding solution‐phase transformation.

This comparison revealed a pronounced advantage for the solid‐state protocol: for methyl 2‐phenyl‐2‐(*p*‐tolylamino)acetate, ball milling delivered 89% yield within 90 min, whereas the analogous solution‐phase reaction reached only 58% after 20 h.

Rhodium complexes have also enabled valuable transfer‐hydrogenation chemistry under mechanochemical conditions, as demonstrated by Ito and coworkers in the peripheral‐selective reduction of poorly soluble polycyclic aromatic hydrocarbons (PAHs) [222].

**SCHEME 40:**
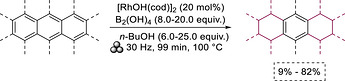
Mechanochemical peripheral hydrogenation of polycyclic aromatic hydrocarbons using a rhodium catalyst, adapted from Ref. [222].

The protocol avoids both gaseous hydrogen and Pd/C [223, 224] by employing B_2_(OH)_4_ as hydrogen surrogate. In the presence of [RhOH(cod)]_2_ and *n*‐BuOH as liquid additive, partial reduction of a range of PAHs was achieved mechanochemically at 100°C, maintained by external heating with a heat gun (Scheme 40). Solubility studies revealed aggregation‐induced emission (AIE) in THF/H_2_O mixtures, with a quantum yield as high as 72% at a 90:10 solvent ratio.

#### 2.2.4 Palladium Mechanocatalysis: A Versatile Platform for C─C and C─Heteroatom Bond Construction

Palladium catalysis remains central to C–C and C–heteroatom bond formation, from Suzuki [225], Negishi [226], Buchwald–Hartwig [227, 228], Mizoroki–Heck [229, 230], and Stille couplings [231] to carbonylation chemistry. Mechanochemical variants show that solvent minimization can coincide with faster kinetics, altered selectivity, safer surrogate chemistry, and simpler operation.

##### 2.2.4.1 Synthesis of Palladium Complexes

**SCHEME 41:**
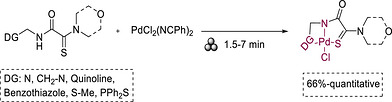
Mechanochemical synthesis of Pd pincer complexes derived from functionalized thioamide ligands, as reported in Ref. [232].

Palladium pincer complexes, whose rigid tridentate frameworks confer high stability and tunable reactivity, have also been prepared and deployed under mechanochemical conditions. In this context, Aleksanyan et al. provided an important mechanochemical entry to pincer complexes derived from functionalized thioamides [232]. While pyridine‐based ligands initially yielded only nonmetalated pincer‐type species, simple grinding of quinoline‐based and *S*,*N*,*S* ligands with PdCl_2_(NCPh)_2_ led directly to the desired palladium complexes. Importantly, subsequent milling experiments performed in an agate vessel with agate balls showed that, for pyridine‐containing systems, mechanochemical synthesis outperformed both manual grinding and the corresponding solution‐based route, highlighting the enabling role of mechanical activation in pincer complex assembly (Scheme 41).

The pure thioanisole‐containing palladium complex formed in just 60 s, an outcome plausibly enabled by the rapid appearance of a low‐melting eutectic phase after only 30 s of grinding.

##### 2.2.4.2 Carbonylation via Solid CO Surrogates

**SCHEME 42:**
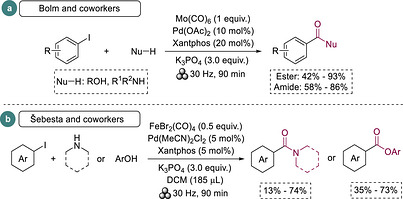
Mechanochemical in situ generated Pd‐complex for carbonylation reactions using metal carbonyls as solid CO surrogates: (a) conditions by Bolm and coworkers [237], (b) by Šebesta and coworkers [238].

While carbonylation chemistry is among the most powerful tools in synthesis, its classical execution remains closely tied to the use of toxic carbon monoxide under pressure [233, 234, 235]. Mechanochemistry reconfigures this paradigm with considerable elegance by replacing gaseous CO with bench‐stable solid carbonyl surrogates [236]. In a seminal study, Bolm and coworkers translated this concept into practice through a palladium‐catalyzed carbonylation of aryl iodides with alcohols under ball‐milling conditions, using Mo(CO)_6_ as the carbon monoxide source [237]. Under an in situ‐generated Pd(OAc)_2_/XantPhos catalytic manifold, room‐temperature milling with Mo(CO)_6_ and K_3_PO_4_ promoted rapid transfer of the carbonyl fragment to palladium. Pressure‐monitoring experiments detected no accumulation of free CO, strongly supporting a mechanism in which carbonyl incorporation occurs without the involvement of gaseous carbon monoxide as a discrete intermediate. An analogous conceptual advance was later realized by Šebesta and coworkers, who used FeBr_2_(CO)_4_ as a solid carbonyl donor for the mechanochemical palladium‐catalyzed synthesis of esters and carboxamides [238]. These studies redefine carbonylation as a safer solid‐surrogate process under milling conditions (Scheme 42).

##### 2.2.4.3 Sustainable Buchwald‐Hartwig Aminations

**SCHEME 43:**
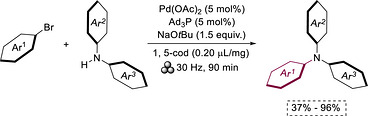
Mechanochemically in situ‐generated Pd‐complex for sustainable Buchwald‐Hartwig amination at room temperature, as reported in Ref. [241].

Palladium‐catalysed C─N bond formation has long attracted sustained interest, driven by the pervasive importance of nitrogen‐containing frameworks in pharmaceutical design and organic optoelectronics [239]. The first mechanochemical Pd‐catalysed C─N bond‐forming reaction conducted under ambient air was reported by Su and coworkers, establishing the conceptual viability of this transformation under solvent‐free conditions [240]. A landmark advance was subsequently achieved by Kubota et al., who overcame a critical limitation of earlier protocols, namely, their dependence on liquid reagents, by developing an air‐stable Buchwald–Hartwig amination of solid amines with aryl bromides under mechanochemical conditions, employing the sterically demanding Ad_3_P ligand (Scheme 43) [241].

The choice of milling media proved decisive: zirconia and agate vessels failed to deliver sufficient mechanical energy for productive coupling, whereas stainless steel jars enabled the reaction to proceed to full conversion, underscoring the nontrivial role of the milling environment in governing reactivity.

Subsequently, Ito and coworkers extended this protocol for the synthesis of *N*‐arylcarbazoles, utilising a combination of Pd(OAc)_2_ and (*t*Bu)_3_P^.^HBF_4_ at elevated temperatures (internal temperature of 125°C) [242].

**SCHEME 44:**
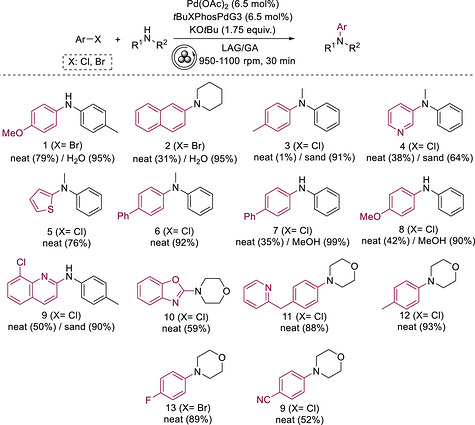
In situ generated Pd‐complex for sustainable Buchwald‐Hartwig amination in a planetary mill, as reported by Geneste and coworkers [243].

The protocol provided efficient access to N‐arylcarbazoles inaccessible by solution‐phase methods, highlighting the enabling value of solid‐state coupling for poorly compatible substrates. A further expansion of scope was reported by Geneste and coworkers, who transposed the coupling protocol to a planetary mill and demonstrated that the combination of Pd(OAc)_2_ with *t*BuXPhosPdG3 could catalyse C─N bond formation with precise reaction control through judicious use of LAG and grinding auxiliaries (Scheme 44) [243].

Notably, the attenuated heat generation characteristic of planetary motion enabled a reduction in catalyst loading without eroding efficiency, a practically significant finding for large‐scale applications. The cumulative mechanochemical toolkit for Buchwald–Hartwig amination was further enriched by the contributions of Bihel and coworkers, whose investigations continued to broaden the synthetic reach of this transformation [244].

##### 2.2.4.4 Advanced Borylation and Suzuki–Miyaura Strategies

**SCHEME 45:**
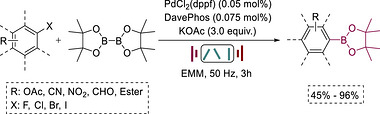
EMM‐promoted palladium‐catalysed borylation of aryl bromides with B_2_pin_2_ under solvent‐free conditions, as reported in Ref. [247].

The electromagnetic mill (EMM) [245], in which an alternating electromagnetic field drives small ferromagnetic bodies inside a stationary vessel, has begun to reveal distinctive synthetic potential [246]. In a noteworthy example, Liu and coworkers exploited the EMM for the palladium‐catalyzed borylation of aryl bromides with B_2_pin_2_ at room temperature, using [Pd(dppf)Cl_2_] and DavePhos as the catalytic system (Scheme 45) [247]. In contrast to the high‐temperature mixer‐mill protocol reported by Kubota et al. [248], the EMM platform enabled efficient borylation without external heating. It outperformed earlier batch processes that required prolonged reaction times at 110°C [249, 250]. Particularly revealing was the complete lack of product formation under planetary ball‐milling conditions. This result underscores how profoundly reaction outcome can depend on the specific mode of mechanical agitation. SEM analysis supported this interpretation, pointing to finer particle‐size distributions and more efficient mixing under EMM conditions.

**SCHEME 46:**
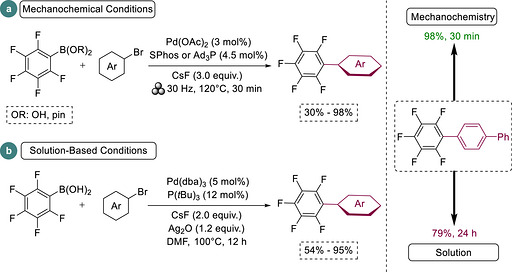
In situ generated Pd‐complex for Suzuki‐Miyaura coupling of polyfluorinated arylborons: (a) using mechanochemical conditions [251], (b) using solution‐based conditions [252].

A particularly elegant example was reported by Takahashi et al., who developed a high‐temperature mechanochemical Suzuki–Miyaura coupling of polyfluorinated arylboron reagents using Pd(OAc)_2_ in combination with Ad_3_P or SPhos (Scheme 46) [251]. By operating under solid‐state conditions, the authors bypassed the need for the stoichiometric Ag_2_O required in solution‐phase protocols and enabled efficient coupling of otherwise weakly nucleophilic polyfluorinated boron partners [252].

**SCHEME 47:**
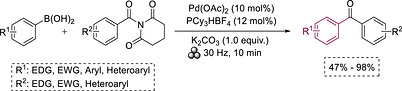
Mechanochemical in situ generated Pd‐complex for Suzuki‐Miyaura coupling using *N*‐acyl‐glutarimides as reported in Ref. [254].

This reactivity manifold was subsequently broadened by Szostak and coworkers, who extended the strategy first to *N*‐acyl glutarimides and then to acyl chlorides as unconventional electrophilic coupling partners (Scheme 47) [253, 254]. Further technological refinement was later achieved by Liu and coworkers through translation of the Suzuki–Miyaura reaction to the EMM platform, where effective coupling could be accomplished with palladium loadings as low as 0.05 mol% using [Pd(dppf)Cl_2_] and DavePhos, even for substrates of limited solubility [255]. The broader synthetic reach of this platform was then illustrated in several directions: Ito and coworkers accessed otherwise intractable oligothiophene derivatives of potential optoelectronic interest by exploiting removable silyl solubilising groups [256], Lamaty and coworkers devised a one‐pot two‐step iodination/Suzuki sequence to furnish C4‐arylated sydnones [257], and Šebesta and coworkers finally extended the coupling manifold to thianthrenium salts and boronic acids under mechanochemical conditions [258].

##### 2.2.4.5 Sustainable Functionalization: Cyanation, Sulfonamides, and C‒C Couplings

Yet classical cyanation chemistry has long been burdened by the use of toxic cyanide sources such as CuCN and Zn(CN)_2_, which generate substantial quantities of metal‐containing waste [259, 260], and by the well‐known tendency of cyanide to poison palladium catalysts by strong coordination [261]. A major conceptual advance was therefore provided by the introduction of K_4_[Fe(CN)_6_] as a comparatively benign and operationally convenient cyanide source under mild conditions [262].

**SCHEME 48:**
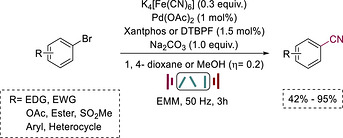
Mechanochemical in situ generated Pd‐complex for cyanation using K_4_[Fe(CN)_6_] as a cyanation agent [263].

Liu and coworkers translated this advance into the mechanochemical domain, reporting an EMM‐based Pd‐catalyzed cyanation of aryl halides in which K_4_[Fe(CN)_6_] serves as the sole cyanide donor (Scheme 48) [263]. Comparative experiments unambiguously demonstrated the indispensability of the EMM platform: both classical ball milling and solution‐phase protocols at room temperature failed to yield any detectable product, underscoring the distinctive energy‐transfer characteristics of the EMM as essential to catalytic competence.

**SCHEME 49:**
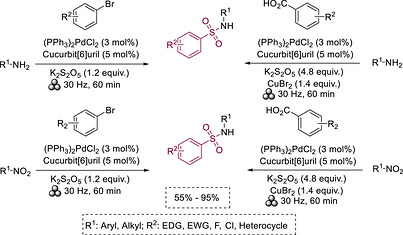
In situ generated Pd‐complex for mechanochemical synthesis of sulfonamides using K_2_S_2_O_5_ as an SO_2_ surrogate, as reported in Ref. [264].

The mechanochemical pursuit of sustainable sulfonamide synthesis was advanced by Iaroshenko and coworkers, who developed a (PPh_3_)_2_PdCl_2_/cucurbit[6]uril‐catalyzed protocol for the coupling of amines with aryl bromides using K_2_S_2_O_5_ as a solid SO_2_ surrogate (Scheme 49) [264].

A complementary protocol replaced aryl bromides with carboxylic acids, incorporating inline decarboxylation at room temperature and avoiding the high temperatures and CuBr_2_ required in solution. SO_2_ also behaved noninnocently as a reductant, enabling conversion of nitro compounds to sulfonamides.

**SCHEME 50:**
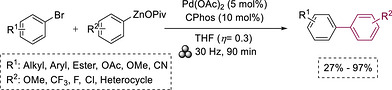
In situ generated Pd‐complex for mechanochemical Negishi‐coupling using aryl bromides and zinc pivalate reagents as reported in Ref. [267].

Inspired by the foundational mechanochemical studies of Ito and coworkers [265] and Knochel and coworkers [266], Šebesta and coworkers reported a mechanochemical Negishi‐coupling protocol employing a Pd(OAc)_2_/CPhos system with aryl bromides and zinc pivalate reagents (Scheme 50) [267], subsequently augmented by a one‐pot, two‐step variant incorporating the in situ generation of Reformatsky enolates [268]. Van Bonn and coworkers addressed oxidative esterification of alcohols [269], while Schnürch and coworkers demonstrated mechanochemical enantiocontrol in Tsuji–Trost allylation, achieving 52% *ee* with (*R*)‐SEGPHOS as the chiral ligand [270]. Liu and coworkers further demonstrated the EMM platform in both Sonogashira [271] and Heck [272, 273] coupling manifolds.

**SCHEME 51:**
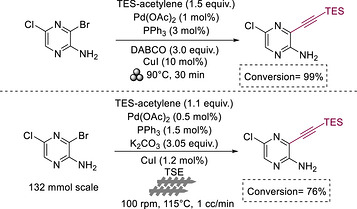
In situ generated Pd‐complex for mechanochemical Sonogashira coupling using ball mills and TSE as reported in Ref. [274].

Speight, Hastings, and coworkers confronted this “translational gap” directly by developing a mechanochemical Sonogashira coupling of aryl bromides with acetylene derivatives, mediated by a Pd(OAc)_2_/PPh_3_ system, initially validated in a mixer mill at 90°C and subsequently transposed to a twin‐screw extruder (TSE) for continuous operation (Scheme 51) [274].

Although the translation was far from trivial, productive and uniform product formation in the TSE required systematic redesign of the screw configuration and an increase in processing temperature to 115°C, underscoring that the physical parameters governing reactivity in batch milling and continuous extrusion are not directly interchangeable. The study underscores that mechanochemical scale‐up is not a direct translation of milling conditions but a process‐engineering problem that requires redesigning energy input, residence time, and material transport.

##### 2.2.4.6 Mechanocatalysis Using Commercial Palladium Complexes

**SCHEME 52:**
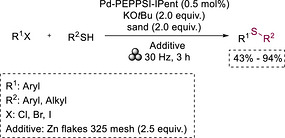
Synthesis of sulfides as reported in Ref. [126].

Browne and coworkers demonstrated the direct use of commercial Pd‐PEPPSI‐IPent in mechanochemical C–S coupling of aromatic and aliphatic thiols with iodoarenes using KO*t*Bu and sand as grinding auxiliary (Scheme 52) [126]. Metallic zinc suppressed inactive disulfide formation in selected cases. Control reactions in 1,4‐dioxane gave only 4%–5% yield after 24 h under air or N_2_, whereas milling proceeded efficiently without bulk solvent or inert atmosphere. The superiority of the solid‐state approach over conventional solution‐phase conditions was unambiguously established by control experiments conducted in 1,4‐dioxane: reactions carried out under air or under N_2_ for 24 h returned yields of only 4% and 5%, respectively, against which the mechanochemical protocol, requiring neither inert atmosphere nor bulk solvent, stands in stark contrast.

Overall, palladium mechanochemistry now spans catalyst activation, reductant management, safer surrogate chemistry, enantioselective variants, telescoped sequences, low catalyst loadings, and scalable processing.

#### 2.2.5 Silver Complexes in Mechanochemical CO_2_ Fixation and Deuterium Incorporation

Silver‐mediated mechanocatalysis remains comparatively underexplored, despite the broad relevance of Ag complexes in cyclisations, alkynylations, rearrangements, aldol reactions [275, 276, 277, 278], and alkene difunctionalization [276, 279].

**SCHEME 53:**
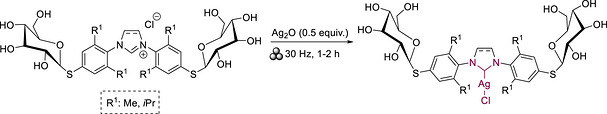
Mechanochemical synthesis of NHC‐Ag complexes, as reported in Ref. [280].

These species are commonly synthesized by combining silver salts with appropriate ligands, leading to distinctive geometries and reactivities that are dictated by the ligand environment and the silver oxidation state. In a notable contribution, Messaoudi and coworkers reported the mechanochemical synthesis of silver complexes featuring NHC carbohydrate ligands derived from thioglycosides (Scheme 53) [280]. Owing to the low solubility of these NHC systems, the reaction was conducted by grinding the ligand with Ag_2_O, affording the target complexes as verified by HR‐MAS ^1^H and ^13^C NMR spectroscopy. The resulting compounds exhibited promising biological activity, reducing the viability of human colon carcinoma HCT‐116 cells to 41%–64% of their original value.

**SCHEME 54:**
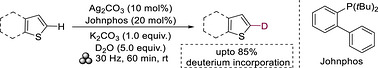
Mechanochemical in situ generated Ag‐complex for deuteration of heteroarenes using D_2_O as reported in Ref. [282].

Gao and coworkers reported mechanochemical deuteration of heteroarenes using an in situ‐generated phosphine‐ligated Ag^I^ complex and D_2_O as the primary deuterium source (Scheme 54) [281, 282]. The method was especially effective for high‐melting polyarenes, where poor solubility limits solution‐phase protocols.

**SCHEME 55:**
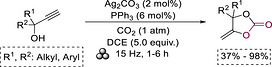
Mechanochemical silver‐mediated CO_2_ fixation, as reported in Ref. [283].

Regarding the application of silver‐catalyzed mechanochemical transformations, Tomita et al. have developed a mechanochemical silver‐catalyzed reaction for the fixation of gaseous CO_2_ to propargyl alcohols to obtain cyclic carbonates, highlighting the ability of mechanocatalysis to activate small gaseous molecules (Scheme 55) [283]. The authors observed a decrease in product yield with increasing milling frequency from 15 Hz to 25 Hz, indicating decomposition of the product under higher mechanochemical impact.

**FIGURE 5:**
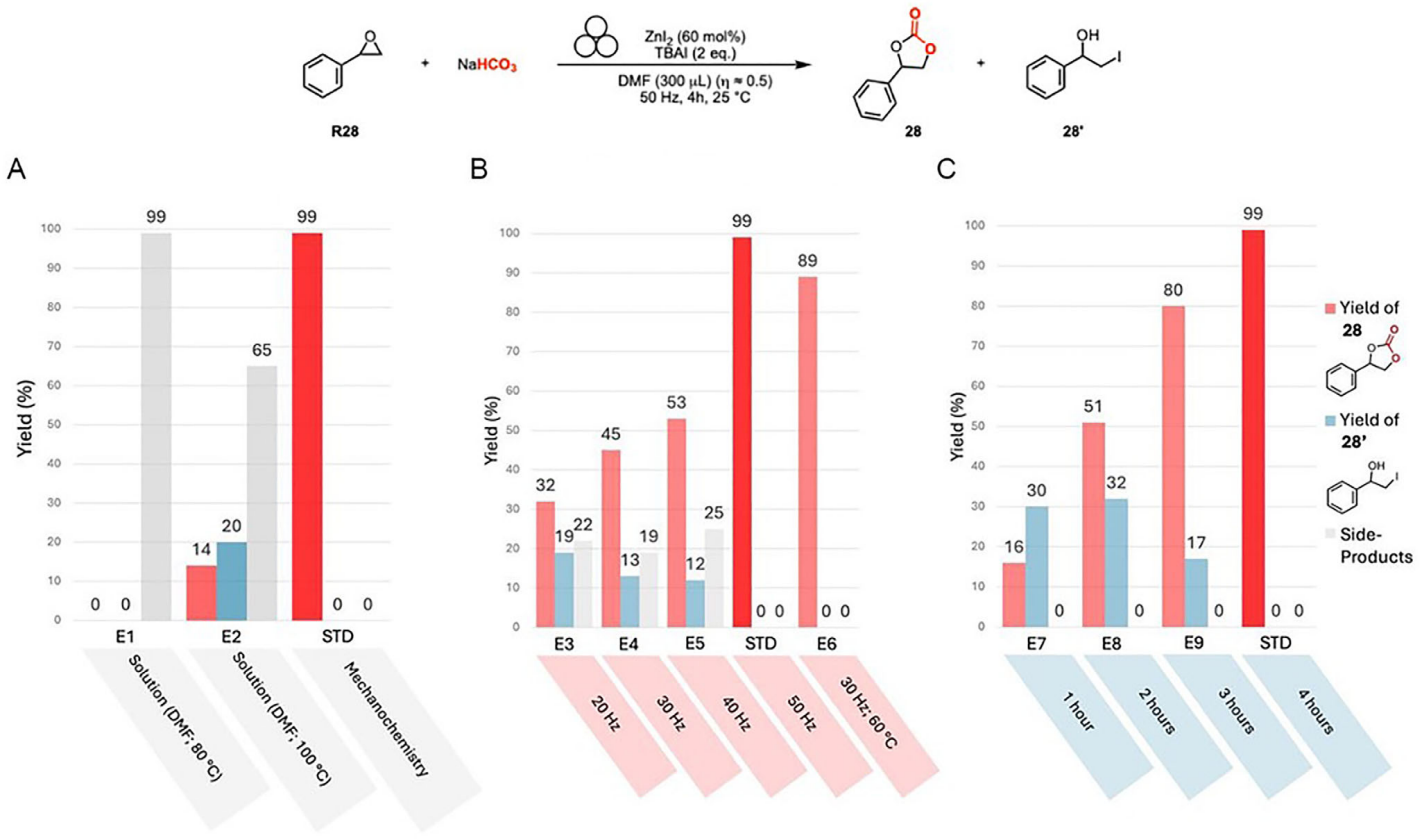
Sodium bicarbonate as a solid CO_2_ donor under mechanochemical conditions for carboxylation reactions. Figure reproduced from Ref. [284].; licensed under CC BY 4.0.

A notable advance in mechanochemical CO_2_ fixation was reported by Mele et al., who demonstrated that NaHCO_3_ can serve as a practical solid surrogate for CO_2_ in ball‐milling‐enabled carboxylation chemistry [284]. In one branch, epoxides were converted into cyclic carbonates via ZnI_2_/TBAI catalysis using dry DMF as LAG additive (Figure 5). This bicarbonate‐to‐carbonate transformation has no precedent in solution, because NaHCO_3_ is poorly compatible with common organic media; milling overcomes this by ensuring solid contact, local CO_2_ release, and immediate epoxide trapping.

In a complementary manifold, the same in situ‐generated CO_2_ was exploited in silver‐mediated carboxylative transformations, further demonstrating that bicarbonate activation under mechanochemical conditions is not restricted to a single substrate class or catalytic platform. Beyond methodological interest, the study extended this strategy to pharmaceutically relevant targets and to a ^13^C‐labelling approach.

#### 2.2.6 Gold Complexes via Mechanochemical Activation

**SCHEME 56:**
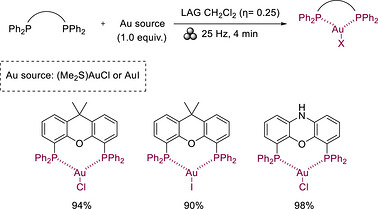
Mechanochemical synthesis of [Au(diphos)] complexes, as reported in Ref. [285].

Gold(I) diphosphine complexes were accessed by milling (Me_2_S)AuCl or AuI with bidentate phosphines in a 1:1 ratio using CH_2_Cl_2_ as LAG additive, affording photofunctional [Au(diphos)X] complexes with strong luminescence and mechanochromic behavior (Scheme 56) [285].

#### 2.2.7 Iridium Mechanocatalysis: Green and Efficient Borylation

**SCHEME 57:**
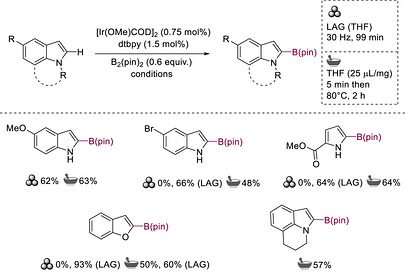
Mechanochemical C–H borylation of heteroarenes with in situ‐generated iridium catalysts under ball‐milling and grind‐and‐heat conditions, as reported in Ref. [289].

Iridium mechanocatalysis is most developed in C–H borylation [286]. Ito and coworkers established early mechanochemical Ir‐catalyzed borylation protocols [287, 288], later extended to heteroarenes and compared with grind‐and‐heat conditions by Pilarski and coworkers (Scheme 57) [289].

With [Ir(OMe)(COD)]_2_/dtbpy and B_2_(pin)_2_, continuous milling and brief grinding followed by heating at 80°C gave comparable heteroarene borylation outcomes. Thus, initial grinding mainly generates an intimately mixed medium, while product formation can proceed by milling or heating; under mixer‐mill conditions, THF was required as LAG additive to maintain rheology.

##### 2.2.7.1 Commercially Available Iridium Complexes in Mechanochemical N‒H Amidation and Cycloaddition

**SCHEME 58:**
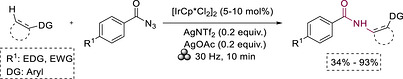
C‒H amidation using acyl azides as reported in Ref. [290].

A critical enabling observation was that acyl azides, when confined within a Teflon milling jar, exhibit markedly greater stability than under solution‐phase conditions, effectively suppressing the competing Curtius rearrangement to the corresponding isocyanates. This side pathway would otherwise erode both yield and selectivity. Capitalizing on this mechanochemically induced stabilization, the authors used acyl azides to mediate C–H amidation of arenes bearing synthetically versatile directing groups, including *N*‐tert‐butylbenzamides and quinoline derivatives [290]. Optimal performance was achieved with [Cp*IrCl_2_]_2_ as the precatalyst, AgNTf_2_ to generate the highly reactive cationic iridium species responsible for nitrene transfer, and AgOAc as the terminal base, collectively enabling efficient amidation across a broad substrate portfolio (Scheme 58). While the solid‐state protocol delivered yields broadly comparable to those of solution‐phase C–H amidation for unfunctionalized substrates, its extension to the synthesis of unsymmetrical ureas revealed a decisive mechanochemical advantage, both in terms of reaction performance and overall sustainability metrics.

**SCHEME 59:**
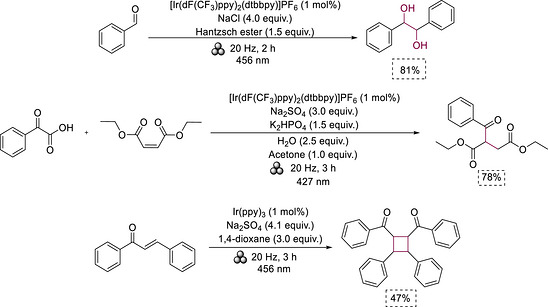
Use of Ir catalysts for mechanophotoredox catalysis, as reported in Ref. [291].

Zysman‐Colman and coworkers exploited this property to pioneer a photomechanocatalytic platform integrating visible‐light iridium photoredox catalysis with mechanochemical activation, demonstrating its applicability across three structurally distinct transformation classes: pinacol couplings, decarboxylative alkylations, and [2+2] cycloadditions (Scheme 59) [291]. In each case, the solid‐state protocol provided a synthetically efficient alternative to solution chemistry, with markedly shortened reaction times.

## 3 Critical Perspectives: Reproducibility, Speciation, and Scale‐Up

Mechanochemistry has matured into a distinct platform for preparing and deploying transition‐metal catalysts, but its broader adoption still requires greater confidence, predictability, and transferability.

### 3.1 Milling‐Media Attrition and Metal Contamination

A major practical limitation of mechanocatalysis is metal contamination from jars and milling balls. High‐energy collisions release Fe, Cr, and Ni into reaction mixtures, which may be acceptable in discovery studies but problematic for pharmaceutical purity standards [292]. Attrition can also perturb sensitive metal centres, complicate catalyst recovery, and impose additional purification. RAM offers a complementary solution by eliminating milling media while retaining scalable acoustic activation.

### 3.2 The Black‐Box Problem: Mechanistic Opacity in In Situ‐Generated Systems

A second, and more fundamental, challenge concerns the structural identity of the catalytically active species under milling conditions. This problem is especially acute for in situ‐generated complexes, a strategy that is synthetically attractive precisely because it bypasses isolation, handling, and pre‐characterisation of catalyst precursors. Under such conditions, however, the coordination environment of the metal within the milling vessel is often inaccessible to conventional analytical methods. Endpoint measurements may confirm conversion, but they rarely resolve the time‐dependent speciation, oxidation‐state evolution, or phase changes that determine catalytic competence. Without reliable knowledge of the active species, rational ligand design, informed optimisation of reaction conditions, and meaningful interlaboratory reproducibility all become substantially more difficult. The development of in situ Raman spectroscopy, synchrotron X‐ray diffraction, and emerging operando solid‐state NMR methodologies offers the most direct route toward addressing this challenge [36].

### 3.3 Stereochemical and Kinetic Control in First‐Row Metal Mechanocatalysis

First‐row metals pose a distinct challenge because SET pathways, radical/ionic competition, and narrow kinetic windows make mechanical parameters decisive for selectivity rather than merely operational. The Fe–salen coupling in Section 2.1.2, which reached 99% ee under milling versus 46% in solution, illustrates the control possible when mechanical activation and LAG are tuned together [85]. Future progress will require kinetic frameworks that integrate impact energy, aging, phase behaviour, and solid‐state stabilisation.

### 3.4 Scalability, From the Milling Jar to Manufacturing

TSE and RAM have narrowed the gap between laboratory demonstration and process‐scale implementation, as illustrated by TSE Suzuki–Miyaura coupling at 5.35 g h^−1^ and the 30 g RAM synthesis of tolbutamide [147, 176].

The principal obstacle is now informational rather than technological: robust correlations between laboratory milling parameters and TSE or large‐scale RAM operating conditions remain sparse. Establishing these relationships as a shared engineering language, analogous to scale‐up methodologies in flow chemistry, is essential for making mechanocatalysis predictive and scalable [197, 293].

## 4 Conclusions

The studies surveyed here show that mechanochemistry has evolved from a solvent‐saving tactic into a primary strategy for constructing and deploying transition‐metal catalysts. Benchmarks including Fe–salen asymmetric coupling up to 99% *ee*, Ni‐catalyzed XEC within minutes, and solid‐state Mo‐mediated N_2_ fixation demonstrate that milling can access reactivity regimes not readily reproduced in solution. The field remains uneven, but its trajectory is now clear: Pd and Ru define the most mature platforms, whereas Ni and Cu are expanding rapidly through XEC, mechano‐redox activation, photomechanochemistry, and RAM. Meanwhile, less explored metals already reveal how mechanical activation can reshape speciation, redox behavior, and catalytic accessibility. The next phase requires fewer demonstrations and greater rigor: routine operando analysis, harmonized reporting of energy delivery and η, and quantitative scale‐up correlations linking milling, TSE, and RAM. Mechanochemistry now has the chemistry; the next task is to make it reproducible, predictive, and deployable at scale.

## Conflicts of Interest

The authors declare no conflicts of interest.

## Data Availability

Data sharing is not applicable to this article as no new data were created or analyzed in this study.
